# Angiotensin-Converting Enzyme Gene I/D Polymorphism Is Associated With Systemic Lupus Erythematosus Susceptibility: An Updated Meta-Analysis and Trial Sequential Analysis

**DOI:** 10.3389/fphys.2018.01793

**Published:** 2018-12-17

**Authors:** Saif Khan, Sajad A. Dar, Raju K. Mandal, Arshad Jawed, Mohd Wahid, Aditya K. Panda, Mohtashim Lohani, B. N. Mishra, Naseem Akhter, Shafiul Haque

**Affiliations:** ^1^Department of Basic Science, College of Dental Sciences, University of Ha'il, Ha'il, Saudi Arabia; ^2^The University College of Medical Sciences and GTB, Guru Teg Bahadur Hospital (University of Delhi), New Delhi, India; ^3^Research and Scientific Studies Unit, College of Nursing & Allied Health Sciences, Jazan University, Jazan, Saudi Arabia; ^4^Department of Bioscience & Bioinformatics, Khallikote University, Berhampur, India; ^5^Department of Biotechnology, Institute of Engineering and Technology, Lucknow, India; ^6^Department of Laboratory Medicine, Faculty of Applied Medical Sciences, Albaha University, Albaha, Saudi Arabia

**Keywords:** genetic variants, polymorphism, *ACE* gene, meta-analysis, genotypic risk, SLE

## Abstract

Angiotensin-converting enzyme (*ACE*) gene is indispensable for endothelial control and vascular tone regulatory systems, usually affected in Systemic Lupus Erythematosus (SLE). *ACE* insertion/deletion (I/D) polymorphism may influence the progress of SLE. Earlier studies have investigated this association without any consistency in results. We performed this meta-analysis to evaluate the precise association between *ACE* I/D polymorphism and SLE susceptibility. The relevant studies were searched until December, 2017 using Medline (PubMed), Google-Scholar and EMBASE search engines. Twenty-five published studies involving 3,308 cases and 4,235 controls were included in this meta-analysis. Statistically significant increased risk was found for allelic (D vs. I: *p* = 0.007; *OR* = 1.202, 95% *CI* = 1.052–1.374), homozygous (DD vs. II: *p* = 0.025; *OR* = 1.347, 95% *CI* = 1.038–1.748), dominant (DD+ID vs. II: *p* = 0.002; *OR* = 1.195, 95% *CI* = 1.070–1.334), and recessive (DD vs. ID+II: *p* = 0.023; *OR* = 1.338, 95% *CI* = 1.042–1.718) genetic models. Subgroup analysis stratified by Asian ethnicity revealed significant risk of SLE in allelic (D vs. I: *p* = 0.045; *OR* = 1.238, 95% *CI* = 1.005–1.525) and marginal risk in dominant (DD+ID vs. II: *p* = 0.056; *OR* = 1.192, 95% *CI* = 0.995–1.428) models; whereas, no association was observed for Caucasian and African population. Publication bias was absent. In conclusion, *ACE* I/D polymorphism has significant role in overall SLE risk and it can be exploited as a prognostic marker for early SLE predisposition.

## Introduction

Systemic lupus erythematosus (SLE) is an autoimmune disease often involving multiple organ inflammation. The clinical consequences of SLE are extremely heterogeneous and generally characterized by pathogenic autoantibody formation against the host's nuclear antigens, immune complex deposition, and end-organ damage (Kyttaris et al., 2006). The precise etiology of SLE is still ambiguous. Earlier studies have proposed that a complex interaction of factors involving gene and environment causes genetic alterations thereby play a key role in the development of SLE in genetically susceptible individuals (Tsao, 2003). In spite of noteworthy advances in understanding the pathogenic role of this disease, diagnosis, prognosis, and therapeutic puzzles are still incomplete. A number of genetic loci have recently been highlighted to be having association with susceptibility to SLE by genome-wide association studies (GWAS), gene association studies, and current advanced single nucleotide polymorphism (SNP) studies (Harley et al., 2006; Gregersen and Olsson, 2009; Frangou et al., 2013). These data clearly highlight the complexity of the genetic interactions involved in SLE progression, suggesting the use of SNPs as promising future biomarkers for assessing genetic background of individuals for prognosis of SLE.

The angiotensin-converting enzyme (ACE, kininase II, EC3.74.15.1) is a zinc metalloproteinase converting angiotensin I (Ang I) into angiotensin II (Ang II), an octapeptide acting as a potent vasopressor and stimulator of aldosterone. Ang II also acts as a growth factor, particularly in kidneys, inducing remodeling of tissue, and fibrosis. Contraction of smooth muscle cells and their proliferation is also known to be induced by Ang II, including adhesion of monocytes and platelets (Morrissey and Klahr, 1998; Kasal and Schiffrin, 2012). *ACE* gene consists of 26 exons and 25 introns amassing to nearly 24 kb in size and is situated on the long arm of the chromosome 17. Many polymorphic residues have also been identified in *ACE*; including a widely studied insertion (I) or deletion (D) of a 287-bp fragment on intron 16 (Rigat et al., 1990), causing three possible genotypes—II, ID, and DD. The genotype DD carrying individuals have 2-fold (higher) levels of *ACE* in serum when compared to the individuals with II genotype. Whereas, ID heterozygotes show a moderate activity while homozygote for I allele reveals the least ACE activity (Sayed-tabatabaei et al., 2006). Therefore, it is possible that this I/D polymorphism may be involved in vascular immunity and SLE pathogenesis. Considering its important role in SLE development, several case-control studies have recently examined the effect of *ACE* I/D polymorphism on the risk of SLE in different populations. However, due to lack of consistency, their results remained inconclusive (Guan et al., 1997; Sato et al., 1998; Tassiulas et al., 1998; Akai et al., 1999; Pullmann et al., 1999; Molad et al., 2000; Kaufman et al., 2001; Prkacin et al., 2001; Uhm et al., 2002; Douglas et al., 2004; Shin, 2004; El-Shafeey et al., 2005; Saeed et al., 2005; Sprovieri and Sens, 2005; Al-Awadhi et al., 2007; Rabbani et al., 2008; Hussain et al., 2010; Abbas et al., 2012; Gong et al., 2012; Lian et al., 2012; Salimi et al., 2012; Topete-Reyes et al., 2013; Negi et al., 2015; Pradhan et al., 2015; Pitipakorn et al., 2016). The inconsistency in their results may be attributed to their probable small sample sizes with petite statistical power. Studying the association of different polymorphisms with complex diseases always requires large sample size as is recommended in a recent study (Burton et al., 2009).

A meta-analysis, as a statistical tool, can overcome these limitations of single studies with small sample sizes. It combines multiple studies on the same alleles of genes to enhance the statistical power of the analysis and derive more precise and reliable results of the genetic effects. Therefore, we performed this meta-analysis by pooling all the eligible published studies to determine a comprehensive picture of the above said genetic association and understand the role of *ACE* gene polymorphism as a genetic marker for SLE progression. To maintain the overall quality of this study, we assessed the selected studies on Newcastle Ottawa Scale (NOS) for their quality score. Trial Sequential Analysis (TSA) was used to minimize type-I statistical errors like publication bias and random errors, caused generally by sparse data, in order to quantify the statistical reliability of the included data in the meta-analysis with statistical significance threshold.

## Materials and Methods

### Search Strategy and Eligibility of the Relevant Studies

A thorough search of studies on the *ACE* I/D gene polymorphism and its association with SLE susceptibility was conducted in PubMed (Medline), Google Scholar and EMBASE. An update limit of December 2017 was applied. The search terms were as follows: “*ACE*” “angiotensin converting enzyme” OR “*ACE*” (polymorphism OR mutation OR variant) in combination with “systemic lupus erythematosus” OR “SLE” (susceptibility OR risk). The focus of the search was human studies only. The titles and abstracts of the all the searched articles were read for initial evaluation, and only studies fulfilling the eligibility criteria were retrieved and used in this meta-analysis. The reference lists of the selected studies was also inspected manually for other pertinent articles.

### Study Selection Criteria

**Figure 1:**
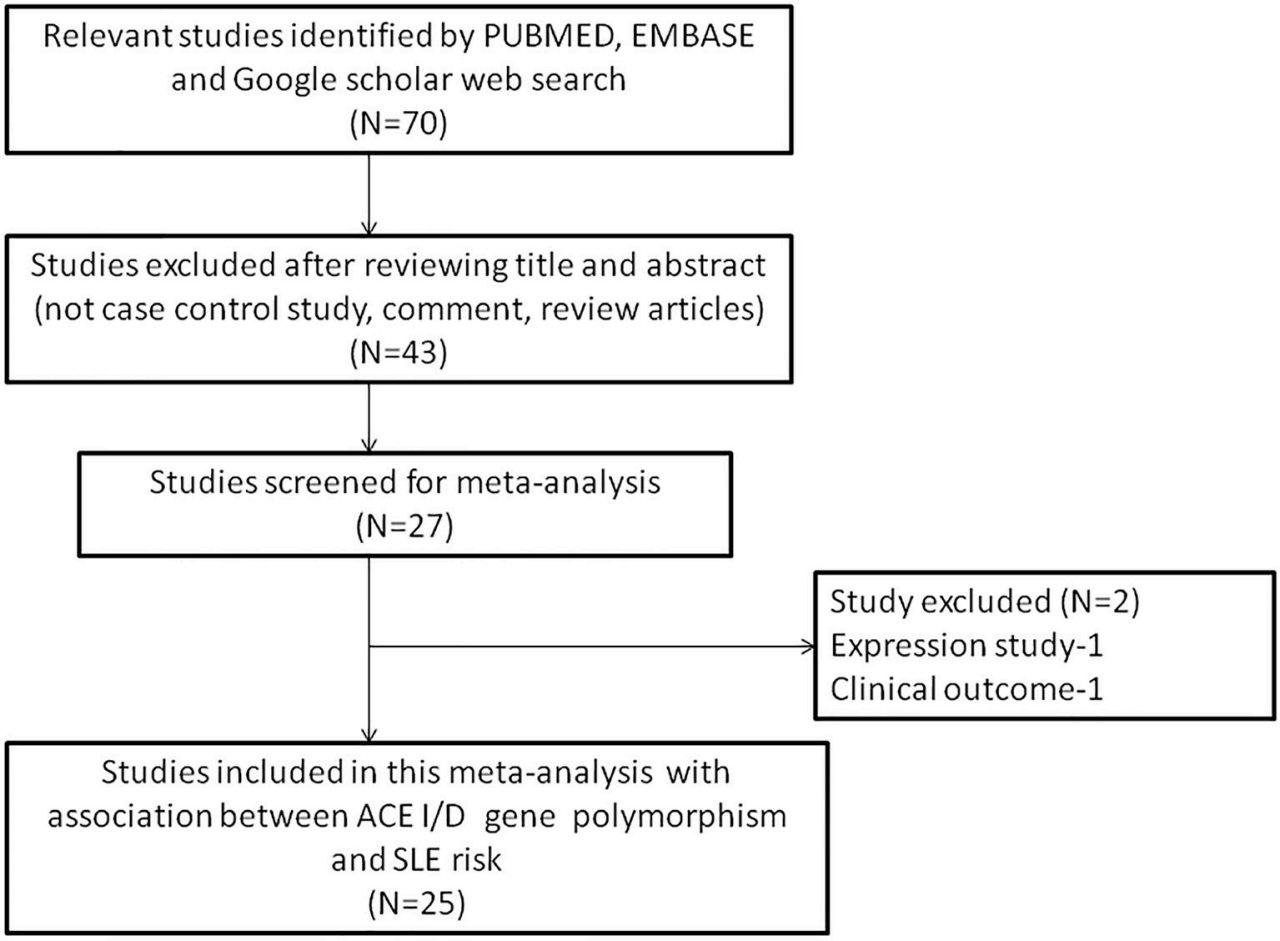
PRISMA 2009 Flow-diagram showing the identification and selection process (inclusion/exclusion) of the pertinent studies for the present meta-analysis.

The following criteria was used for selection of studies for this meta-analysis: (a) the study must be an evaluation of the association between *ACE* I/D gene polymorphism and SLE susceptibility, (b) the study must have a case-control design, (c) the study must have enrolled well diagnosed SLE patients and normal healthy controls, (d) the study must have genotypic frequency data available for both patients and controls, (e) the study must be published in English. In case the data for same patient population was reported in more than one publication, the most recent and complete publication was considered for this meta-analysis. The exclusion criteria for the studies were: studies with overlapping data; studies reporting data for patient population only; studies where genotypic frequency data was not available; and review articles. All information regarding selection of the studies is depicted as PRISMA 2009 Flow-Diagram (Figure 1).

### Extraction of the Data From Selected Studies

Two investigators (RKM & SAD), independently, extracted and summarized the data from each retrieved study by following the standard procedure. The data collection form was designed and used to collect the data to ensure accuracy while following stringent inclusion/exclusion criteria as described above. The attributes extracted and summarized from the selected articles were: first author's name; publication year; origin country; total number of patients and controls included; study type; association status; genotyping method used; and genotype frequency of patients and controls. Discrepancy, if observed, in the data collected from the selected studies by the two investigators was settled by open discussion in presence of SH (an adjudicator) in order to reach a final consensus.

### Newcastle-Ottawa Scale (NOS) Criteria for Quality Assessment

The NOS criteria (Stang, 2010) was used to assess the methodological quality of the selected studies. This was again done separately by two independent investigators (RKM & SAD). Three major aspects are included in the NOS criteria—(i) selection of subjects (0–4 points); (ii) comparability of subjects (0–2 points); (iii) clinical outcomes (0–3 points). Studies getting 5 or more than 5 points or stars were considered as of moderate to high or good quality (Hu et al., 2015). The disagreement, if any, in assigning the points or stars to the study, by the two investigators, was resolved in consultation with the adjudicator SH.

### Statistical Analysis

The statistical analyses was performed using Comprehensive Meta-Analysis (CMA) V2 software program (Biostat, USA). The *p* < 0.05 was considered as statistically significant. All the *p*-values were two sided. Crude odds ratios (ORs) and corresponding 95% confidence intervals (CIs) were computed to assess the association intensity between the *ACE* I/D gene polymorphism and SLE susceptibility. The allele contrast, log-additive, dominant, and recessive model pooled ORs were estimated (Woolf, 1955). Chi-square based Q-test was used to perform heterogeneity assumption across the eligible studies (Wu and Li, 1999), and heterogeneity was considered significant when *p* < 0.05. A fixed effect model was used when *p* > 0.05 (Mantel and Haenszel, 1959); and a random effect model was used when *p* < 0.05 (DerSimonian and Laird, 1986). For efficient testing of the heterogeneity, I^2^ statistics was also employed (Higgins et al., 2003). Chi-square test was used to measure Hardy-Weinberg equilibrium (HWE) in the control population. Egger's linear regression test was used to estimate funnel plot asymmetry, a type of linear regression approach on the natural logarithm ORs scale. The *t*-test was used to determine the significance of the intercept; statistically significant publication bias was indicated by *p* < 0.05 (Egger et al., 1997).

### Trial Sequential Analysis (TSA)

In an attempt to include all the eligible trials in this meta-analysis, and to minimize the systematic errors (bias) or random errors by chance, we used a novel statistical TSA tool from Copenhagen Trial Unit, Center for Clinical Intervention Research, Denmark. TSA estimates the required information size and adjusts statistical significance thresholds, and also estimates the power of conclusion (Wetterslev et al., 2008; Brok et al., 2009; Turner et al., 2013). TSA indicates no requirement of further trials if the Z curve crosses the monitoring boundary before the required information size is reached; however, if it does not cross the boundary, further trials becomes necessary. The TSA software program, version 0.9 (http://www.ctu.dk/tsa/) was used for TSA analysis.

### Cochran-Armitage (CA) Statistics

We determined Cochran-Armitage (CA) statistics for each of the (29) cases considered in our study. CA trend test is the most popular association analysis for determining genetic associations (Sasieni, 1997).

CA trend test statistic (T) is given by the following equation

(1) $$\begin{array}{l} T=\overset{3}{\underset{i=1}{\sum }}M_{i}\left(N_{y}X_{i}-N_{x}Y_{i}\right) \end{array}$$

Where

For a codominant or additive model, *M*_1_ = 0; *M*_2_ = 1;*M*_3_ = 2.

For a Dominant Model, *M*_1_ = 0; *M*_2_ = 1; *M*_3_ = 1.

For a Recessive model, *M*_1_ = 0; *M*_2_ = 1; *M*_3_ = 2.

Here, *N*_*y*_ is the total number of controls, *N*_*x*_ is the total number of cases, *X*_*i*_ and *Y*_*i*_ represents the number of cases and controls for each of the three (II, ID, and DD) genotypes.

## Results

### Literature Search and Meta-Analysis Databases

During the study search the full-texts of all the articles deemed potentially eligible were retrieved. The study eligibility for inclusion was determined by reviewing the full text of all the articles by the first investigator. The second investigator randomly selected 10% of the articles and reviewed them by the same procedure, independently. A complete agreement was observed between the two investigators regarding inclusion and exclusion of the studies. Following the identification of the final set of the eligible articles, one investigator extracted the relevant data from all the studies, and the other investigator independently re-extracted the data from all the included studies to cross-check this step. Table 1, 2 depict the main characteristics and genotype distribution along with minor allele frequency (MAF) in subjects of all the 25 studies included in this meta-analysis, respectively. In NOS analysis for quality score, more than 95% of all the included studies scored 5 stars or more, except the study of Hussain et al. (2010) which scored only 3 stars. This study was included because it possessed all the basic needful information for its consideration in the analysis and its 3 stars suggested a moderate to good quality (Table 3). The sequential process for identification of the eligible studies for the present meta-analysis followed the pre-set inclusion and exclusion criteria shown in Figure 1 (PRISMA 2009 Flow Diagram).

**Table 1:** Main characteristics of all the studies of *ACE* I/D polymorphism and SLE risk included in this meta-analysis.

**References**	**Country**	**Ethnicity**	**Type of Study**	**Control**	**Cases**	**Main findings**
Pitipakorn et al., 2016	Thailand	Asian	HB	687	187	Reduce risk with DD genotype
Negi et al., 2015	India	Asian	HB	460	300	No risk with any genotype
Pradhan et al., 2015	India	Asian	HB	100	109	Increased risk with DD and ID genotype
Topete-Reyes et al., 2013	Mexico	Caucasian	PB	144	65	No risk with any genotype
Salimi et al., 2012	Iran	Asian	HB	103	106	Increased risk with DD genotype
Gong et al., 2012	China	Asian	HB	320	314	Increased risk with DD genotype
Abbas et al., 2012	Egypt	African	HB	29	50	Increased risk with D allele
Lian et al., 2012	Malaysia	Asian	HB	190	170	Increased risk with ID genotype
Hussain et al., 2010	Pakistan	Asian	PB	61	61	Increased risk with DD genotype
Rabbani et al., 2008	Pakistan	Asian	HB	79	39	No risk with any genotype
Al-Awadhi et al., 2007	Kuwait	Asian	HB	100	92	No risk with any genotype
El-Shafeey et al., 2005	Egypt	African	HB	30	50	No risk with any genotype
Saeed et al., 2005	Pakistan	Asian	HB	79	39	No risk with any genotype
Sprovieri and Sens, 2005	Brazil	Mixed	HB	65	147	No risk with any genotype
Douglas et al., 2004	USA	African	PB	70	140	No risk with any genotype
Douglas et al., 2004	USA	Caucasian	PB	201	85	No risk with any genotype
Shin, 2004	Korea	Asian	HB	171	84	No risk with any genotype
Uhm et al., 2002	Korea	Asian	HB	114	211	No risk with any genotype
Prkacin et al., 2001	Croatia	Caucasian	HB	21	18	Increased risk with DD genotype
Kaufman et al., 2001	USA	African	PB	129	128	Increased risk with D allele
Kaufman et al., 2001	USA	Caucasian	PB	291	206	No risk with any genotype
Kaufman et al., 2001	USA	Other	PB	45	30	No risk with any genotype
Molad et al., 2000	Israel	Caucasian	HB	48	56	No risk with any genotype
Akai et al., 1999	Japan	Asian	HB	100	84	Increased risk
Pullmann et al., 1999	Slovakia	Caucasian	HB	148	101	Increased risk with D allele
Sato et al., 1998	Japan	Asian	HB	100	93	No risk with any genotype
Tassiulas et al., 1998	USA	African	HB	78	78	Reduce risk with DD genotype
Tassiulas et al., 1998	USA	Caucasian	HB	122	121	Reduce risk with DD genotype
Guan et al., 1997	China	Asian	HB	150	144	Increased risk with DD genotype

*HB, hospital based; PB, population based*.

**Table 2:** Genotypic distribution of *ACE* I/D gene polymorphism in studies included in this meta-analysis.

**References**	**Controls**				**Cases**				**HWE**
	**Genotype**			**Minor allele**	**Genotype**			**Minor allele**	
	**II**	**ID**	**DD**	**MAF**	**II**	**ID**	**DD**	**MAF**	***p*-value**
Pitipakorn et al., 2016	318	195	174	0.395	48	52	9	0.321	0.001
Negi et al., 2015	172	206	82	0.402	101	140	59	0.430	0.140
Pradhan et al., 2015	21	57	22	0.505	12	61	36	0.610	0.161
Topete-Reyes et al., 2013	37	75	32	0.482	14	37	14	0.500	0.606
Salimi et al., 2012	42	47	14	0.364	34	45	27	0.466	0.882
Gong et al., 2012	128	157	35	0.354	90	144	80	0.484	0.199
Abbas et al., 2012	0	19	10	0.672	2	17	31	0.790	0.008
Lian et al., 2012	91	60	39	0.363	70	83	17	0.344	0.001
Hussain et al., 2010	6	32	23	0.639	4	3	54	0.909	0.282
Rabbani et al., 2008	27	38	14	0.417	14	14	11	0.461	0.920
Al-Awadhi et al., 2007	14	45	41	0.635	19	36	37	0.597	0.770
El-Shafeey et al., 2005	7	12	11	0.566	5	27	18	0.630	0.309
Saeed et al., 2005	27	38	14	0.417	14	14	11	0.461	0.920
Sprovieri and Sens, 2005	8	39	18	0.576	17	69	61	0.649	0.064
Douglas et al., 2004	13	32	25	0.585	25	66	49	0.585	0.627
Douglas et al., 2004	42	95	64	0.554	13	44	28	0.588	0.539
Shin, 2004	62	82	27	0.397	29	44	11	0.392	0.989
Uhm et al., 2002	39	57	18	0.407	82	87	42	0.405	0.707
Prkacin et al., 2001	5	11	5	0.501	4	5	9	0.638	0.827
Kaufman et al., 2001	22	60	47	0.596	22	41	65	0.667	0.703
Kaufman et al., 2001	62	144	85	0.539	54	91	61	0.516	0.944
Kaufman et al., 2001	11	24	10	0.488	9	12	9	0.5	0.652
Molad et al., 2000	7	15	26	0.697	3	20	33	0.767	0.072
Akai et al., 1999	35	50	15	0.400	42	33	9	0.303	0.676
Pullmann et al., 1999	37	68	43	0.520	13	49	39	0.628	0.333
Sato et al., 1998	48	39	13	0.325	33	46	14	0.397	0.266
Tassiulas et al., 1998	8	27	43	0.724	10	44	24	0.589	0.239
Tassiulas et al., 1998	14	63	45	0.627	10	59	52	0.673	0.250
Guan et al., 1997	82	59	9	0.256	58	44	42	0.444	0.705

**Table 3:** Quality assessment conducted according to the Newcastle-Ottawa Scale for all the studies included in the present meta-analysis.

**References**	**Quality indicators**		
	**Selection**	**Comparability**	**Exposure**
Pitipakorn et al., 2016	***	*	**
Negi et al., 2015	**	*	***
Pradhan et al., 2015	***	*	**
Topete-Reyes et al., 2013	***	*	***
Salimi et al., 2012	**	*	**
Gong et al., 2012	***	*	**
Abbas et al., 2012	***	*	**
Lian et al., 2012	**	*	**
Hussain et al., 2010	*	*	*
Rabbani et al., 2008	**	*	**
Al-Awadhi et al., 2007	***	*	***
El-Shafeey et al., 2005	**	*	**
Saeed et al., 2005	**	*	**
Sprovieri and Sens, 2005	***	*	**
Douglas et al., 2004	****	*	***
Shin, 2004	***	*	***
Uhm et al., 2002	**	*	***
Prkacin et al., 2001	**	*	**
Kaufman et al., 2001	*	*	***
Molad et al., 2000	****	*	***
Akai et al., 1999	**	*	**
Pullmann et al., 1999	***	*	**
Sato et al., 1998	**	*	**
Tassiulas et al., 1998	***	*	***
Guan et al., 1997	***	*	**

On assessing the quality of the included studies using the Newcastle-Ottawa Scale, all the studies scored five stars or more barring the study by Hussain et al. (2010) (despite low score, the study of Hussain et al., 2010 was included because the needful genotypic frequency was available in the article), indicating no bias. Each ^*^ indicates one point scored for each indicator during NOS quality assessment.

### Publication Bias Diagnosis

Both, funnel plot asymmetry and Egger's regression statistics were employed to evaluate the publication bias. A *p* < 0.05 was fixed for the significant publication bias in the present meta-analysis. All the comparison genetic models showed absence of publication bias (*p* > 0.05) (Table 4, Figure SI1).

**Table 4:** Statistics to test publication bias and heterogeneity in meta-analysis: Overall analysis.

**Comparisons**	**Egger's regression analysis**			**Heterogeneity analysis**			**Model used for this meta-analysis**
	**Intercept**	**95% Confidence interval**	***p*-value**	***Q*-value**	**P_**heterogeneity**_**	***I*^**2**^ (%)**	
D vs. I	0.80	−1.35 to 2.96	0.45	92.14	0.001	69.61	Random
DD vs. II	0.21	−1.67 to 2.09	0.81	78.12	0.001	64.16	Random
ID vs. II	−0.73	−1.92 to 0.45	0.21	46.95	0.014	40.36	Random
DD+ID vs. II	−0.11	−1.22 to 1.00	0.83	38.57	0.088	27.40	Fixed
DD vs. ID+II	0.93	−1.80 to 3.68	0.48	119.43	0.001	76.55	Random

### Heterogeneity Evaluation

Heterogeneity was tested by *Q*-test and *I*^2^ statistics among the included studies. Four genetic models showed heterogeneity, so the data was synthesized by applying the random effect model (Table 4).

### Association of ACE I/D Polymorphism and Overall SLE Susceptibility

**Figure 2:**
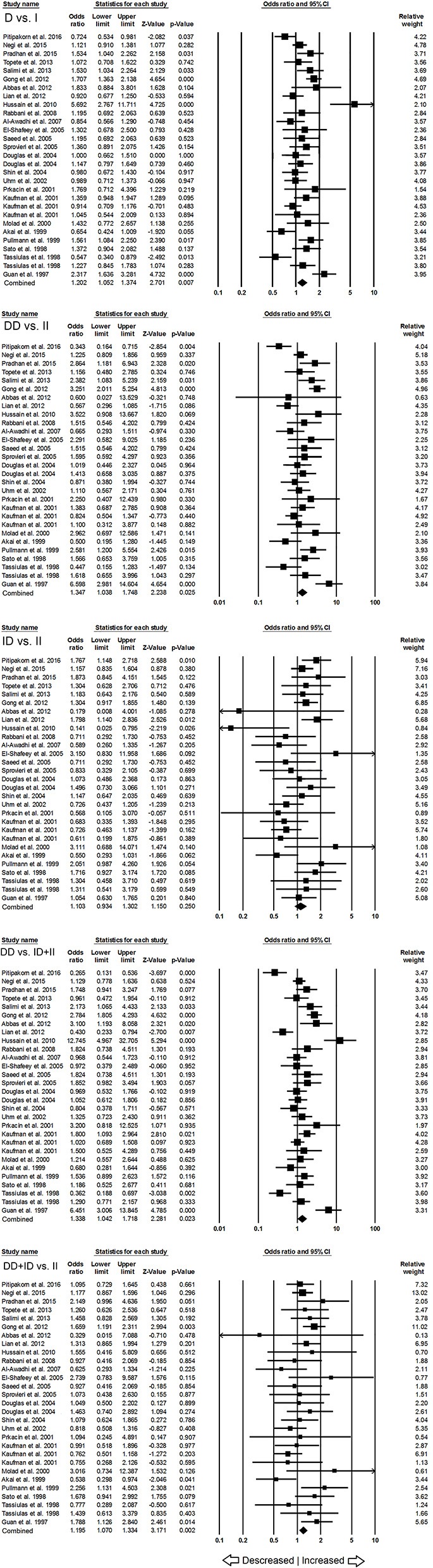
Forest plot of ORs with 95% CI of SLE risk associated with the *ACE* I/D gene polymorphism for the overall population. Black square represents the value of OR and the size of the square indicates the inverse proportion relative to its variance. Horizontal line is the 95% CI of OR.

When pooled together, the number of cases accumulated to 3,308 and controls to 4,235 from all the included studies. The pooled subjects were examined for the precise association between *ACE* I/D polymorphism and overall SLE susceptibility. Overall, the pooled analysis suggests significant increased risk between *ACE* I/D gene polymorphism and overall SLE risk in four genetic models, i.e., allelic (D vs. I: *p* = 0.007; *OR* = 1.202, 95% *CI* = 1.052–1.374), homozygous (DD vs. II: *p* = 0.025; *OR* = 1.347, 95% *CI* = 1.038–1.748), dominant (DD+ID vs. II: *p* = 0.002; *OR* = 1.195, 95% *CI* = 1.070–1.334) and recessive (DD vs. ID+II: *p* = 0.023; *OR* = 1.338, 95% *CI* = 1.042–1.718) (Figure 2). Whereas, heterozygous (ID vs. II: *p* = 0.250; *OR* = 1.103, 95% *CI* = 0.934–1.302) genetic model did not show any SLE risk (Figure 2).

### Association of ACE I/D Polymorphism and SLE Susceptibility in Asian Population

**Figure 3:**
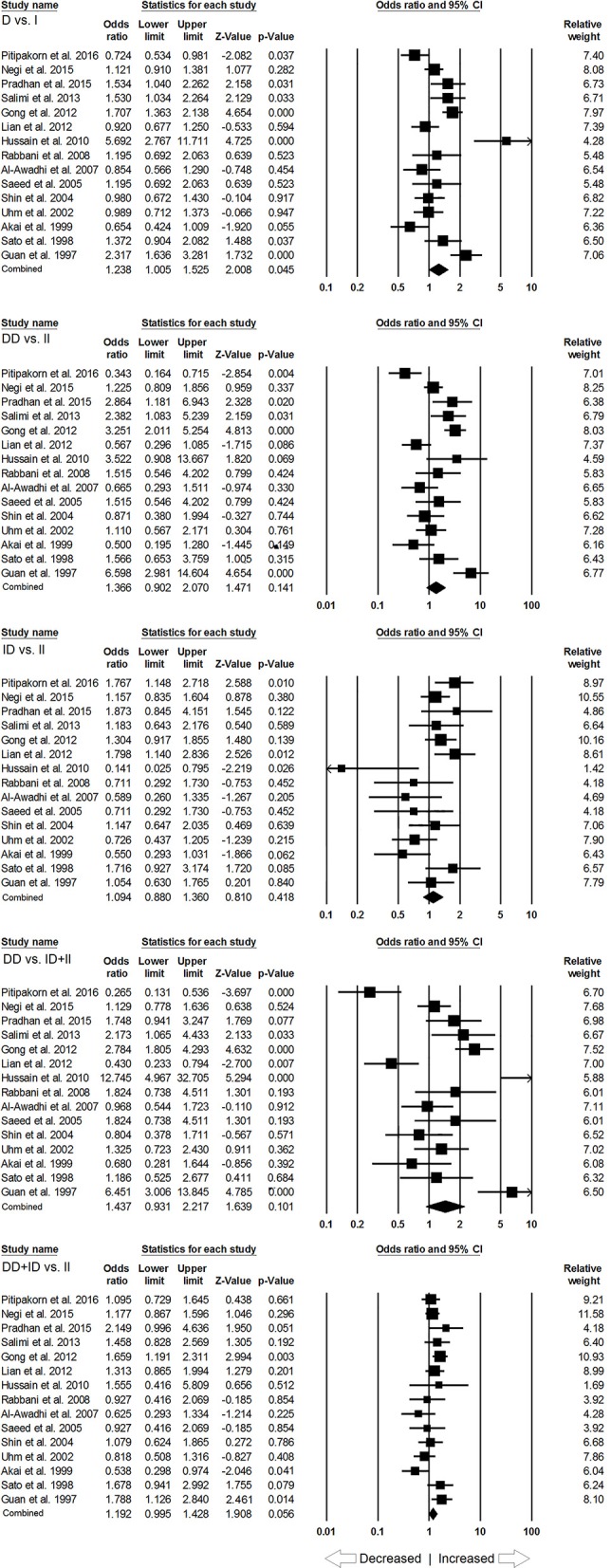
Forest plot of ORs with 95% CI of SLE risk associated with the *ACE* I/D gene polymorphism for the Asian subgroup population. Black square represents the value of OR and the size of the square indicates the inverse proportion relative to its variance. Horizontal line is the 95% CI of OR.

In subgroup analysis of Asian ethnic population, 15 studies involving a total of 2,083 confirmed SLE cases and 2,844 controls were considered for this analysis. Heterogeneity was observed in four genetic models (Figure SI2), so the random effect models were applied to generate ORs and 95% CIs for the synthesis (Table 5). We observed increased risk of SLE development in allelic (D vs. I: *p* = 0.045; *OR* = 1.238, 95% *CI* = 1.005–1.525) genetic model. Similarly, dominant (DD+ID vs. II: *p* = 0.056; *OR* = 1.192, 95% *CI* = 0.995–1.428) genetic model also showed marginal significant risk. But, other genetic models, i.e., homozygous (DD vs. II: *p* = 0.141; *OR* = 1.366, 95% *CI* = 0.902–2.070), heterozygous (ID vs. II: *p* = 0.418; *OR* = 1.094, 95% *CI* = 0.880–1.360) and recessive (DD vs. ID+II: *p* = 0.101; *OR* = 1.437, 95% *CI* = 0.931–2.217) genetic models did not show any increased or decreased risk of SLE (Figure 3).

**Table 5:** Statistics to test publication bias and heterogeneity in the present meta-analysis: Asian population.

**Comparisons**	**Egger's regression analysis**			**Heterogeneity analysis**			**Model used for the meta-analysis**
	**Intercept**	**95% Confidence interval**	***p*-value**	***Q*-value**	**P_**heterogeneity**_**	***I*^**2**^ (%)**	
D vs. I	0.89	−3.36 to 5.15	0.65	70.88	0.001	80.25	Random
DD vs. II	−0.26	−4.39 to 3.87	0.89	63.9	0.001	78.11	Random
ID vs. II	−2.14	−4.23 to−0.06	0.04	30.10	0.007	53.49	Random
DD+ID vs. II	−0.81	−3.03 to 1.40	0.44	24.22	0.043	4.21	Random
DD vs. ID+II	0.83	−4.40 to 6.07	0.73	92.15	0.001	84.80	Random

### Association of ACE I/D Polymorphism and SLE Susceptibility in Caucasian Population

**Figure 4:**
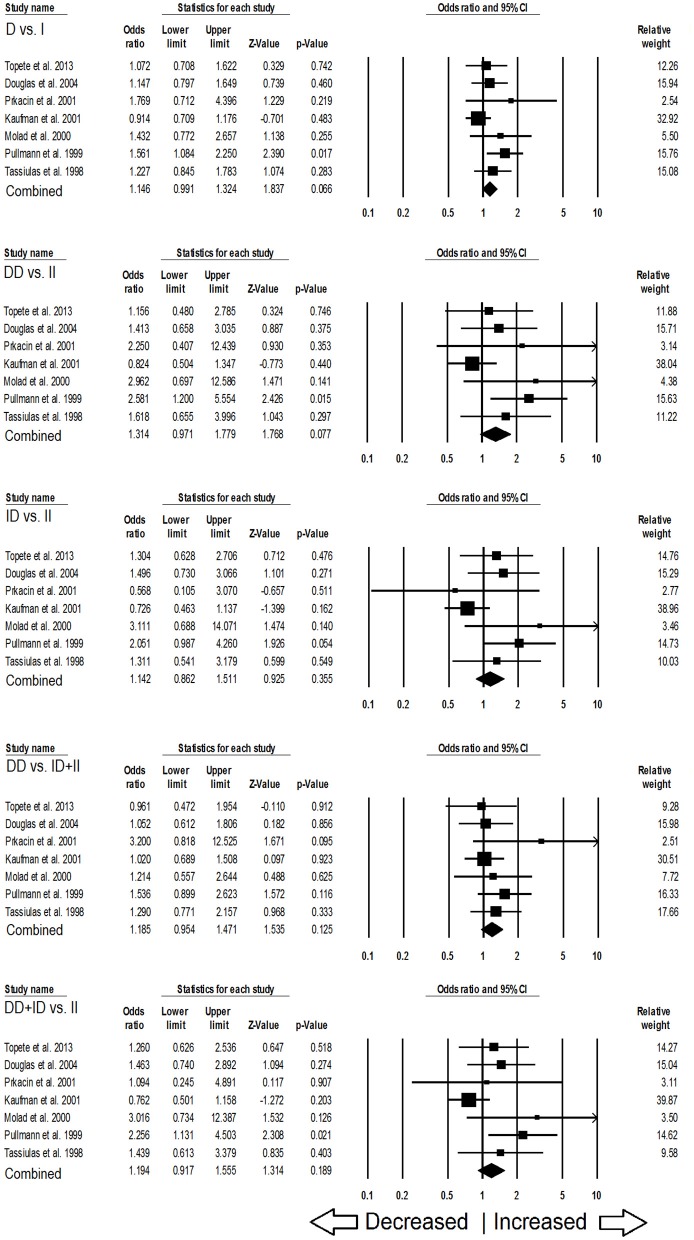
Forest plot of ORs with 95% CI of SLE risk associated with the *ACE* I/D gene polymorphism for the Caucasian subgroup population. Black square represents the value of OR and the size of the square indicates the inverse proportion relative to its variance. Horizontal line is the 95% CI of OR.

In case of subgroup analysis of Caucasian ethnicity population, 7 studies comprising of 652 confirmed SLE cases and 975 controls were included. No heterogeneity was observed in all the genetic models (Figure SI3), so the fixed effect models were applied to generate ORs and 95% CIs (Table 6). After the synthesis, we found that all the genotypic models were not significantly associated with SLE risk, i.e., allelic (D vs. I: *p* = 0.066; *OR* = 1.146, 95% *CI* = 0.991–1.324), homozygous (DD vs. II: *p* = 0.077; *OR* = 1.314, 95% *CI* = 0.971–1.779), heterozygous (ID vs. II: *p* = 0.355; *OR* = 1.142, 95% *CI* = 0.862–1.511), dominant (DD+ID vs. II: *p* = 0.189; *OR* = 1.194, 95% *CI* = 0.917–1.555) and recessive (DD vs. ID+II: *p* = 0.125; *OR* = 1.185, 95% *CI* = 0.954–1.471) genetic models (Figure 4).

**Table 6:** Statistics to test publication bias and heterogeneity in the present meta-analysis: Caucasian population.

**Comparisons**	**Egger's regression analysis**			**Heterogeneity analysis**			**Model used for the meta-analysis**
	**Intercept**	**95% Confidence interval**	***p*-value**	***Q*-value**	**P_**heterogeneity**_**	**I^**2**^ (%)**	
D vs. I	2.16	−0.49 to 4.83	0.09	7.44	0.28	19.39	Fixed
DD vs. II	2.20	−0.28 to 4.69	0.07	8.36	0.21	28.25	Fixed
ID vs. II	1.50	−1.60 to 4.62	0.26	9.49	0.14	36.80	Fixed
DD+ID vs. II	2.06	−0.68 to 4.82	0.11	9.89	0.12	39.35	Fixed
DD vs. ID+II	1.51	−0.77 to 3.80	0.14	4.13	0.65	0.001	Fixed

### Association of ACE I/D Polymorphism and SLE Susceptibility in African Population

**Figure 5:**
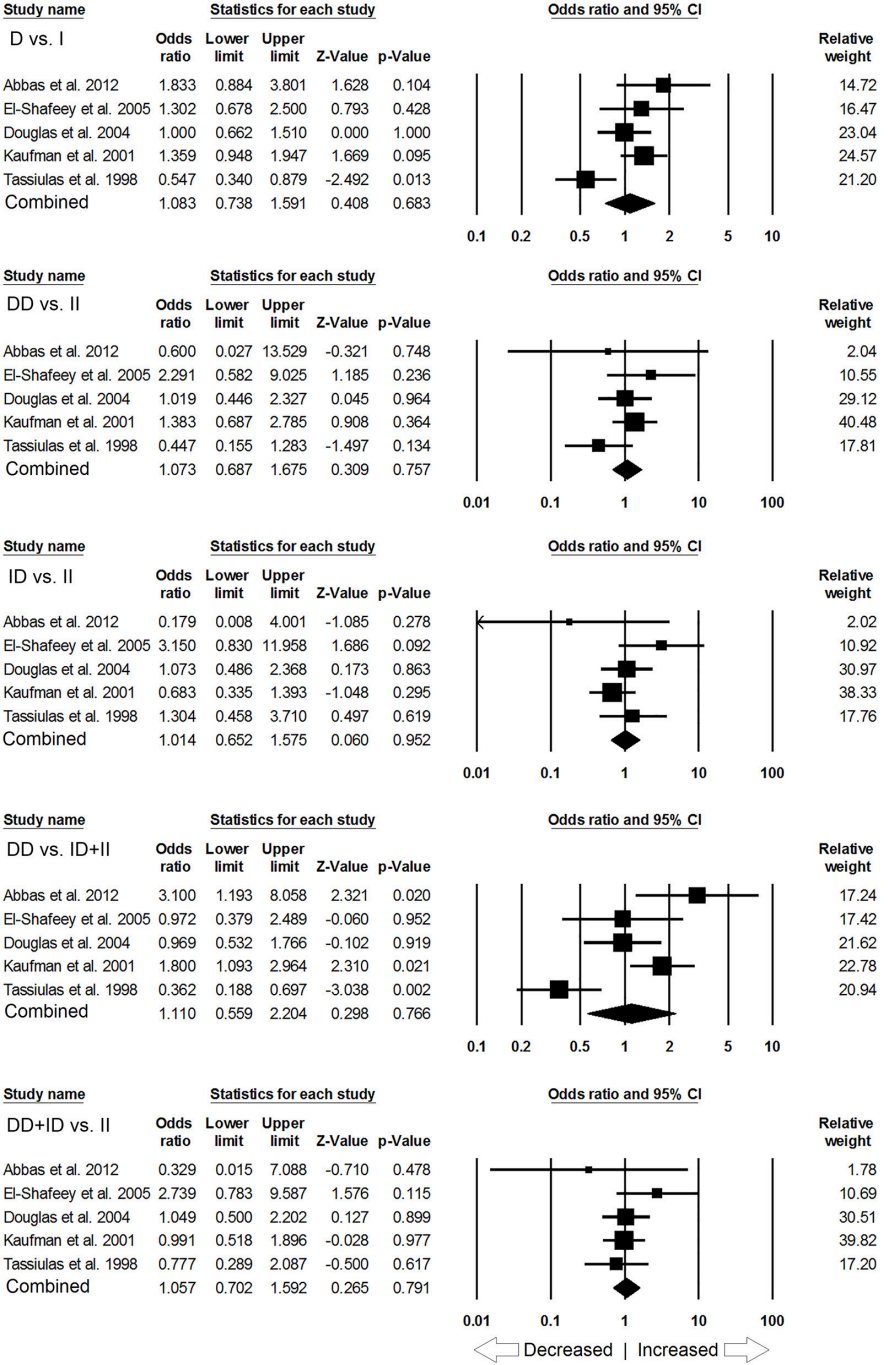
Forest plot of ORs with 95% CI of SLE risk associated with the *ACE* I/D gene polymorphism for the African subgroup population. Black square represents the value of OR and the size of the square indicates the inverse proportion relative to its variance. Horizontal line is the 95% CI of OR.

Likewise, in case of subgroup analysis of African ethnicity population, 5 studies with a total number of 446 confirmed SLE cases and 336 controls were included for the pooled analysis. We observed heterogeneity in two genetic models (Figure SI4), so the random effect models were applied to generate ORs and 95% CIs (Table 7). Also, we found that all the genotypic models were not significantly associated with increased or decreased risk of SLE, i.e., allelic (D vs. I: *p* = 0.683; *OR* = 1.083, 95% *CI* = 0.738–1.591), homozygous (DD vs. II: *p* = 0.757; *OR* = 1.073, 95% *CI* = 0.687–1.675), heterozygous (ID vs. II: *p* = 0.952; *OR* = 1.014, 95% *CI* = 0.652–1.575), dominant (DD+ID vs. II: *p* = 0.791; *OR* = 1.057, 95% *CI* = 0.702–1.592) and recessive (DD vs. ID+II: *p* = 0.766; *OR* = 1.110, 95% *CI* = 0.559–2.204) genetic models (Figure 5).

**Table 7:** Statistics to test publication bias and heterogeneity in the present meta-analysis: African population.

**Comparisons**	**Egger's regression analysis**			**Heterogeneity analysis**			**Model used for the meta-analysis**
	**Intercept**	**95% Confidence interval**	***p*-value**	***Q*-value**	**P_**heterogeneity**_**	**I^**2**^ (%)**	
D vs. I	0.94	−10.20 to 12.10	0.80	11.91	0.018	66.42	Random
DD vs. II	−0.43	−4.90 to 4.03	0.77	4.48	0.345	10.72	Fixed
ID vs. II	0.13	−4.89 to 5.16	0.93	5.39	0.250	25.79	Fixed
DD+ID vs. II	−0.10	−3.85 to 3.64	0.93	3.18	0.527	0.001	Fixed
DD vs. ID+II	0.05	−14.86 to 14.97	0.99	19.54	0.001	79.53	Random

### Sensitivity Analysis

To appraise the effect of an individual study on the overall SLE risk, we performed leave-one-out sensitivity analysis and recomputed the pooled ORs. The estimated pooled ORs calculated after excluding a single study did not show any differences from the primary values. This suggests that the results of sensitivity analysis were stable for overall SLE risk (Figure SI5). Moreover, the estimated pooled ORs also did not show any change in the subgroup analyses (for Asian, Caucasian, and African ethnicities), which suggested that the results of subgroup analyses were robust (Figure SI6–SI8, respectively).

### Trial Sequential Analysis (TSA) of ACE I/D Gene Polymorphism With SLE Risk

**Figure 6:**
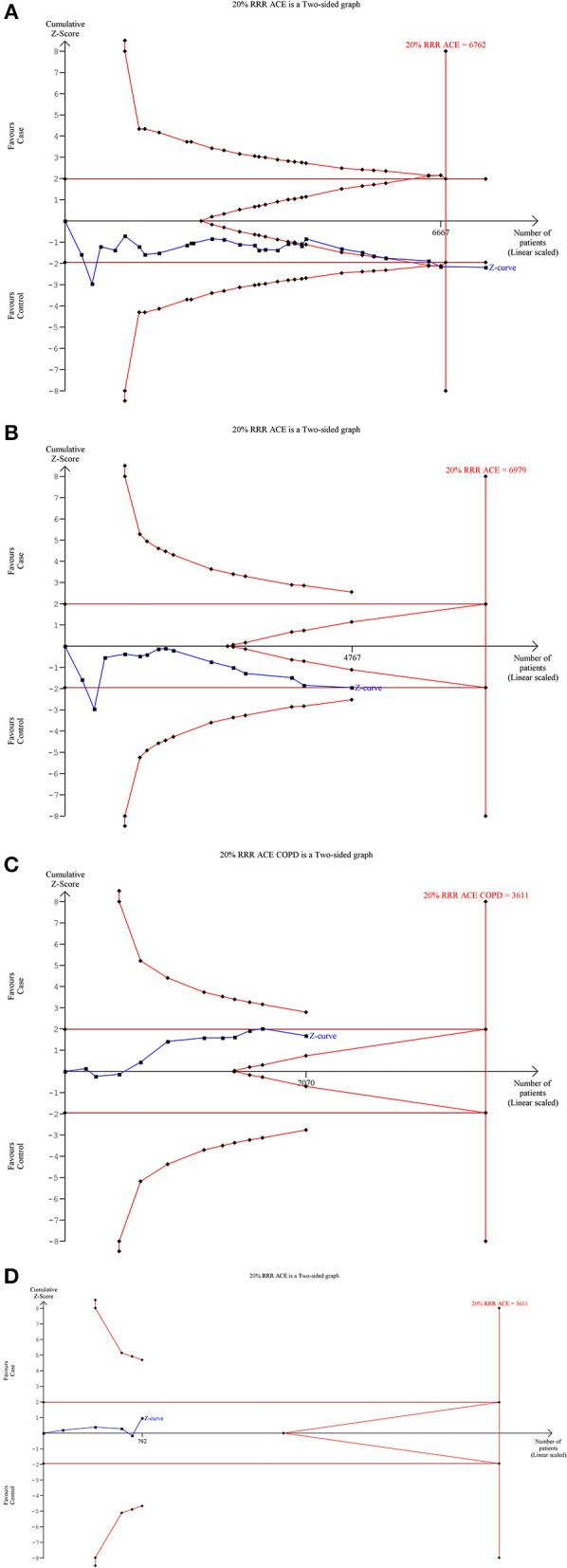
Trial sequence analysis of all the studies on *ACE* I/D gene polymorphism and SLE risk based on dominant genetic model: **(A)** Overall, **(B)** Asian, **(C)** Caucasian, and **(D)** African.

Our TSA analysis depicted that cumulative Z curve crossed the trial monitoring boundary before required information size (6762 subjects) was reached. The dominant model was taken as an example in the TSA analysis, which indicated that *ACE* I/D gene polymorphism is associated with SLE risk and hence no further trials are required (Figure 6A). Subgroup analysis based on ethnicity revealed that Z curve did not cross the trial monitoring boundary before required information size was reached, suggesting insufficiency of cumulative evidence, therefore demanding further trials (Figures 6B–D).

### Cochran-Armitage (CA) and Log Odds Ratio (OR)

A comparison of the results obtained from OR and CA trend test is shown in Table 8. It can be observed that significant association was found mostly in the case of Asian population (eight cases out of total twelve significant associations observed as per CA trend test, Table 8). CA results confirm that more than 53% of Asian association studies have a significant association of I/D polymorphism to SLE. For African Population three out of the total five studies considered in this meta-analysis were found to have significant recessive model association to SLE susceptibility; however more studies are required to state a clear association. The Caucasian ethnicity appears to have no significant association of I/D polymorphism to SLE susceptibility (only one out of seven studies considered in this meta-analysis was found to have a significant dominant association). Table 8 reports the Models, which were found significant as per the log odds ratio and CA trend test. It is evident that in all the cases considered the results from CA and Log odds ratio (OR) are same (column 4 and 5, Table 8). It can be observed that the results from the log odds ratio exactly follow the findings from the CA trend test (detailed *p*-values can be referred from the attached Excel Sheet: Supplementary Material).

**Table 8:** Comparison of Log Odds Ratio (OR) and CA trend test results.

**Study**	**Model@OR^**α**^**	**Model@CA^**α**^**	**Result significance@OR**	**Result** **significance@CA**	**Ethnicity^**β**^**
Pitipakorn et al., 2016	DD vs. ID+II^*^ D vs. I DD vs. II ID vs. II	**Recessive**	**Significant**	**Significant**	**Asian**
Negi et al., 2015	None	None	Insignificant	Insignificant	Insignificant
Pradhan et al., 2015	DD vs. II^*^ D vs. I	**Additive**	**Significant**	**Significant**	**Asian**
Topete-Reyes et al., 2013	None	None	Insignificant	Insignificant	Insignificant
Salimi et al., 2012	DD vs. II^*^ DD vs. ID+II D vs. I	**Recessive** Additive Dominant	**Significant**	**Significant**	**Asian**
Gong et al., 2012	DD vs. II^*^ D vs. I DD vs. ID+II DD+ID vs. II	**Recessive** Additive Dominant	**Significant**	**Significant**	**Asian**
Abbas et al., 2012	DD vs. ID+II^*^	**Recessive**	**Significant**	**Significant**	**African**
Lian et al., 2012	DD vs. ID+II^*^ ID vs. II	**Recessive**	**Significant**	**Significant**	**Asian**
Hussain et al., 2010	DD vs. ID+II^*^ D vs. I ID vs. II	**Recessive** Dominant	**Significant**	**Significant**	**Asian**
Rabbani et al., 2008	None	None	Insignificant	Insignificant	Insignificant
Al-Awadhi et al., 2007	None	None	Insignificant	Insignificant	Insignificant
El-Shafeey et al., 2005	None	None	Insignificant	Insignificant	Insignificant
Saeed et al., 2005	None	None	Insignificant	Insignificant	Insignificant
Sprovieri and Sens, 2005	None	None	Insignificant	Insignificant	Insignificant
Douglas et al., 2004	None	None	Insignificant	Insignificant	Insignificant
Douglas et al., 2004	None	None	Insignificant	Insignificant	Insignificant
Shin, 2004	None	None	Insignificant	Insignificant	Insignificant
Uhm et al., 2002	None	None	Insignificant	Insignificant	Insignificant
Prkacin et al., 2001	None	None	Insignificant	Insignificant	Insignificant
Kaufman et al., 2001	DD vs. ID+II^*^	**Recessive**	**Significant**	**Significant**	**African**
Kaufman et al., 2001	None	None	Insignificant	Insignificant	Insignificant
Kaufman et al., 2001	None	None	Insignificant	Insignificant	Insignificant
Molad et al., 2000	None	None	Insignificant	Insignificant	Insignificant
Akai et al., 1999	DD + ID vs. II^*^	**Dominant**	**Significant**	**Significant**	**Asian**
Pullmann et al., 1999	**DD vs**. II^*^ D vs. I DD + ID vs. II	**Dominant** **Additive**	**Significant**	**Significant**	**Caucasian**
Sato et al., 1998	None	None	Insignificant	Insignificant	Insignificant
Tassiulas et al., 1998	**DD vs**. ID+II^*^ D vs. I	**Recessive** Additive	**Significant**	**Significant**	**African**
Tassiulas et al., 1998	None	None	Insignificant	Insignificant	Insignificant
Guan et al., 1997	DD vs. ID+II^*^ D vs. I DD vs. II DD + ID vs. II	**Recessive** Additive Dominant	**Significant**	**Significant**	**Asian**
**Asian population only**	D vs. I^*^ DD vs. II DD + ID vs. II DD vs. ID+II ID vs. II	**Additive** Dominant Recessive	**Significant**	**Significant**	**Asian**
**All populations (Combined)**	D vs. I^*^ DD vs. II DD + ID vs. II DD vs. ID+II ID vs. II	**Additive Dominant Recessive**	**Significant**	**Significant**	**ALL**

α *Only significant Models (p < 0.05) are reported*.

* *Model with lowest p-value is reported in Bold letters. All the other models (if found significant i.e., p < 0.05) are listed in descending order of p-value, i.e., one with the lowest p-value at the top*.

β *Ethnicities with significant p-values are reported only*.

## Discussion

Earlier studies have reported that individual's SLE susceptibility often present a variety of symptoms that pose difficulty in the disease diagnosis by the physicians, leading to a consequent delay in the onset of the treatment. Host factors, including genetic polymorphisms implicated in autoimmune diseases, might have interpreted this divergence. Thus, there is an urgent need of the identification of the genetic biomarkers that are responsible for the onset and progress of SLE. Nowadays, the current research focus is based on the role of genetic susceptibility in autoimmune disease progression or development.

Renin—angiotensin system (RAS) regulates the arterial blood pressure at both levels—systemic and tissue, and contributes to the immunological responses arising during various phases of nephropathy evolution (Egido, 1996). The major cause of late stage morbidity and mortality in SLE patients is ischemic heart disease (IHD) (Urowitz et al., 1976; Starfelt et al., 1992). It looks evident that *ACE* plays an important role in SLE etiology by affecting the immune responses and vascular changes. Angiotensin II converted by *ACE* is a potent pro-inflammatory modulator for immune responses in the renal tissues and shows robust potential in mediating the development and progression of renal disease during SLE (Suzuki et al., 2000; Taal et al., 2000). As the *ACE* gene has different SNP sites, and research studies have shown that SNPs are capable of changing the structure of the genome and influence protein expression and function that leads to increased risk of different autoimmune diseases, for e.g., SLE. It is possible that Ins/Del (I/D) genotype/allele might confer susceptibility in SLE patients. Individual studies generally have low statistical power to detect the risk, owing to their small sample sizes. Therefore, it is more judicious to estimate the precise relationship of *ACE* I/D gene polymorphism to understand the contribution of this polymorphism in overall SLE risk.

Meta-analysis, as a statistical strategy, is capable of reducing the pernicious effect of the stochastic processes on studies reporting the false-positive and false-negative associations by pooling the sample size from similar studies. Using the meta-analysis strategy, we in this study pooled the data from all the 25 eligible case-control studies and observed that the individuals carrying D allele are at the higher risk of developing SLE when compared to individuals carrying the wild I allele. This supportive evidence (i.e., the results of meta-analysis) was further confirmed by the TSA and comparison with CA trend test statistics, which authenticated the association of *ACE* I/D polymorphism with an increased SLE risk. These results confirm that *ACE* I/D genetic variant may interfere with its expression level and plays a pivotal role in the progression of SLE. Earlier studies have reported that circulating *ACE* levels vary greatly between individuals and is extremely determined genetically. *ACE* I/D polymorphism could alter the circulation of ACE levels. It has been found that individuals carrying DD genotype had 2-folds higher circulating ACE levels and lowest among the II genotype carrying individuals (Cambien et al., 1988; Alhenc-Gelas et al., 1991).

Above studies have reported higher serum ACE levels in SLE patients. However, the interruption of the renin angiotensin with ACE inhibitors or angiotensin receptor blockers (ARBs) is recommended as first line adjuvant therapy for the patients with lupus nephritis for proteinuria (Bertsias et al., 2012). Earlier studies on lupus patients and experimental studies on lupus-prone mice suggested that renin angiotensin system inhibition reduces glomerular injury and proteinuria along with transforming growth factor beta, a major mediator of renal fibrosis (De Albuquerque et al., 2004; Tselios et al., 2014). Animal studies have also enlightened the inflammatory components of renin angiotensin and the potential benefits of angiotensin blockade in reducing or eliminating the inflammation in lupus nephritis (Teplitsky et al., 2006). Lupus nephritis patients not using ACE inhibitor have shown association with increased carotid atherosclerosis (Ravenell et al., 2012). Earlier studies have shown that ACE inhibitor delays the occurrence of renal involvement and stabilizes the disease activity in SLE patients (Duran-Barragan et al., 2008). Therefore, these agents may be used in clinical improvement of arterial hypertension and proteinuria in SLE patients.

The present meta-analysis gives a preliminary overview of the involvement of *ACE* I/D gene polymorphism in SLE etiology and sheds valuable insight on its pathogenesis. Therefore, a better understanding of *ACE* related genetic, epigenetic, environmental, and clinical factors may add to the effective prevention methods for SLE treatment. The defects in the immune-surveillance complex pathway may shed light on new therapeutic targets for SLE.

During the subgroup analysis of *ACE* I/D polymorphism and SLE risk in each ethnic group, the pooled analysis demonstrated that *ACE* I/D polymorphism is significantly associated with SLE risk in Asian population. However, this polymorphism has no role of SLE risk in Caucasian and African population. Since the overall number of studies in non-Asian population is less, it may be possible that the ethnic subgroup analyses of Caucasian and African population may have delineated ambiguous outcomes. Hence, larger studies with bigger sample size from Caucasian and African population are warranted to explore the precise association in this subgroup ethnicity analysis.

The polygenic nature of SLE etiology, and diversity in role of *ACE* I/D gene polymorphism in developing SLE risk renders implication of a single gene variant for risk of developing this complex autoimmune disease due to clinical heterogeneity and acquired genetic alterations in SLE.

In comparison with the previously published meta-analyses (Zhou et al., 2012; Lee et al., 2013), our study included all the eligible studies which were missed somehow in the previous meta-analyses and also added some new eligible studies providing the largest sample size for *ACE* I/D polymorphism and SLE risk. Some advantageous features of the present meta-analysis are application of NOS scale for stringent quality assessment where majority of studies showed good quality in terms of sample size, genotypes, and inclusion of patients and healthy controls; adoption of strict search and pre-set selection procedure for the inclusion of the studies; recruitment of more studies (missed in earlier meta-analyses) for increased statistical power and robust conclusion; well exploration of the methodological issues generally occurring in pooled analysis (for e.g., publication bias and sensitivity) which further confirmed the reliability and validity of the present study; and reduction of type I error rate by Trial sequential analysis.

Despite the obvious strengths of this meta-analysis (large sample size and TSA implementation), this study also suffers with several limitations, which must be stated and addressed in the future studies. First, the result of overall population may be biased, perhaps owing to slight over-representation of Asian population. Second, the result of subgroup analysis especially for Caucasian and African, it may be possible that the observed result could be partly due to modest number of studies included. Third, there was a significant heterogeneity in some of the pooled analysis, which may affect the meta-analysis outcome. Fourth, unadjusted estimates form the basis of this meta-analysis and studies published in English language only were included. Unpublished data and ongoing studies were not searched and included. Further, studies reporting negative findings are less likely to be published, hence causing publication bias thereby bringing increase of the associations. Fifth, due to lack of sufficient data, we were not able to study lupus nephritis individually in relationship with *ACE* allele in this meta-analysis. Sixth, we failed to study the gender associations due to limited data. We hope that peer or future researchers will attempt to study the association between SLE susceptibility and gender specification genotype of *ACE* I/D in the near future.

In conclusion, the pooled results of independent association studies by meta-analysis confirmed statistically significant association between *ACE* I/D gene polymorphism and SLE susceptibility. Individuals carrying allele “D” of the *ACE* I/D polymorphism had greater risk of SLE. The findings will advance our understanding of the role of *ACE* I/D genetic variant and assist in identifying the “at-risk” individuals. Our results also provide a solid foundation for future genetics studies to focus on *ACE* related phenotypes and integrative network modules analysis to clarify the potential role of *ACE* genetic variants in SLE risk. Moreover, more meta-analysis with larger sample size should also be encouraged in future to understand the molecular mechanism of *ACE* gene with SLE development and to verify the current findings reported in this manuscript.

## Author Contributions

SK, SD, RM, MW, AJ, AP, ML BM, NA, and SH conceived and designed the study and experiments. SD, SK, RM, MW, AP, NA, and SH performed the experiments. SD, SK, RM, AJ, AP, ML, BM, and NA analyzed the data. AJ, MW, AP, BM, ML, NA, SD, and SH contributed reagents, materials, analysis tools. SD, SK, RM, AP, and SH wrote the paper. All authors reviewed and approved the manuscript.

### Conflict of Interest Statement

The authors declare that the research was conducted in the absence of any commercial or financial relationships that could be construed as a potential conflict of interest.
