# Supplementary material for: Angiotensin-Converting Enzyme Gene I/D Polymorphism Is Associated With Systemic Lupus Erythematosus Susceptibility: An Updated Meta-Analysis and Trial Sequential Analysis
Source: Front Physiol. 2018 Dec 17;9:1793. doi: 10.3389/fphys.2018.01793 (PMC6305102; doi:10.3389/fphys.2018.01793)
Supplement: Supplementary file 1 [file Data_Sheet_1.PDF]

Angiotensin-Converting Enzyme Gene I/D Polymorphism is Associated with Systemic Lupus Erythematosus Susceptibility: An Updated Meta-analysis and Trial Sequential Analysis

Authors' names in order of their authorship
Saif Khan[1#], Sajad A. Dar[2,3#], Raju K. Mandal[3#], Arshad Jawed[3], Mohd Wahid[3], Aditya K. Panda[4], Mohtashim Lohani[3], B.N. Mishra[5], Naseem Akhter[6], Shafiul Haque[3]*

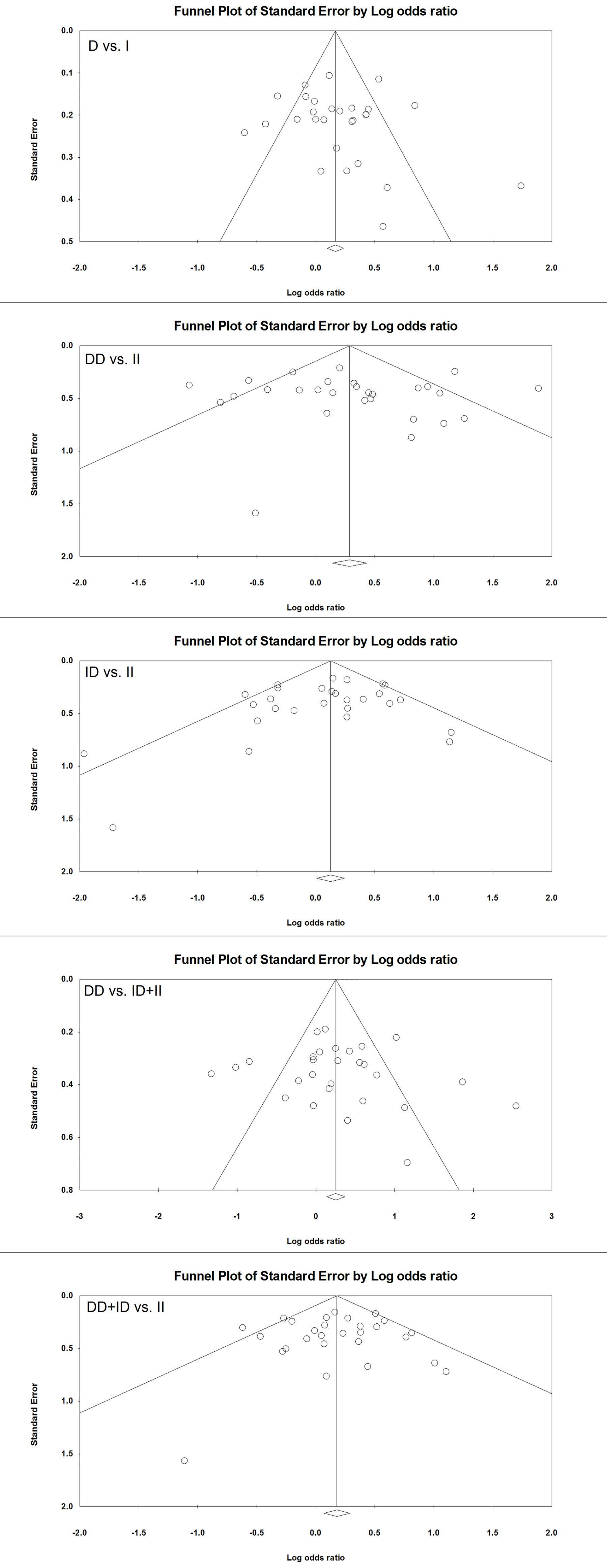

Figure SI1: Assessment of publication bias shown with Funnel plots in studies assaying odds of SLE associated with the ACE I/D polymorphism for overall analysis (Odds ratio against standard error in different genetic models).

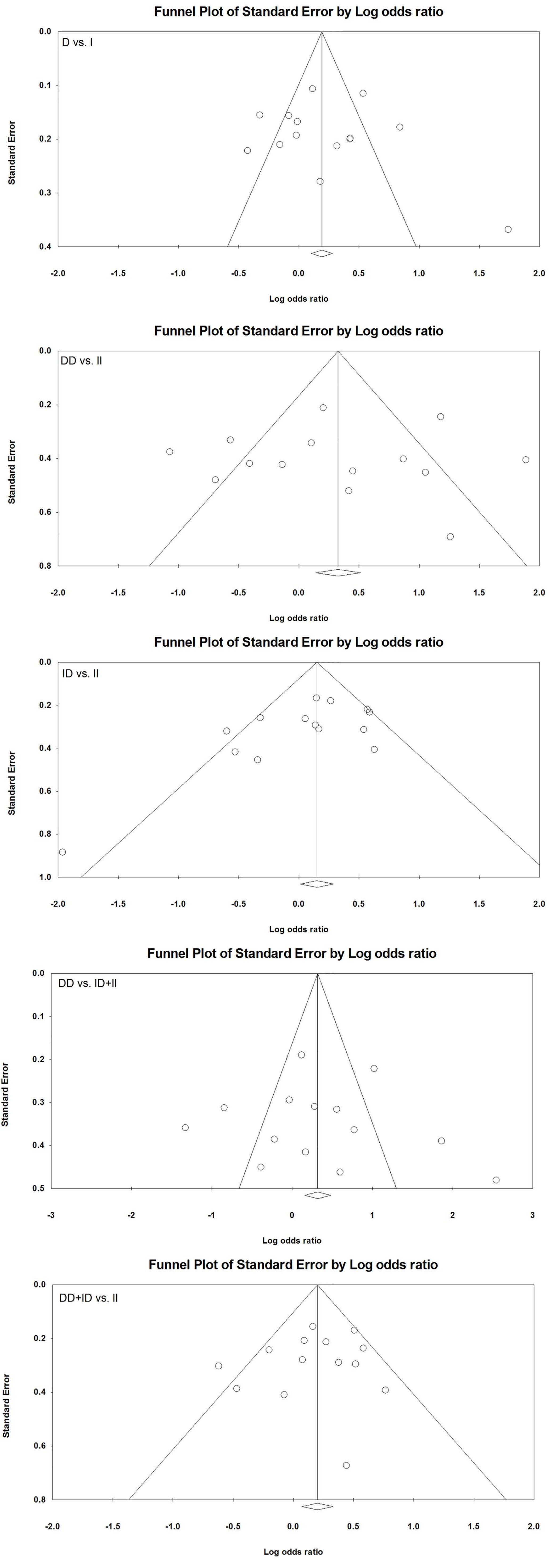

Figure SI2: Assessment of publication bias shown with Funnel plots in studies assaying odds of SLE associated with the ACE I/D polymorphism for sub-group (Asian ethnicity population) analysis (Odds ratio against standard error in different genetic models).

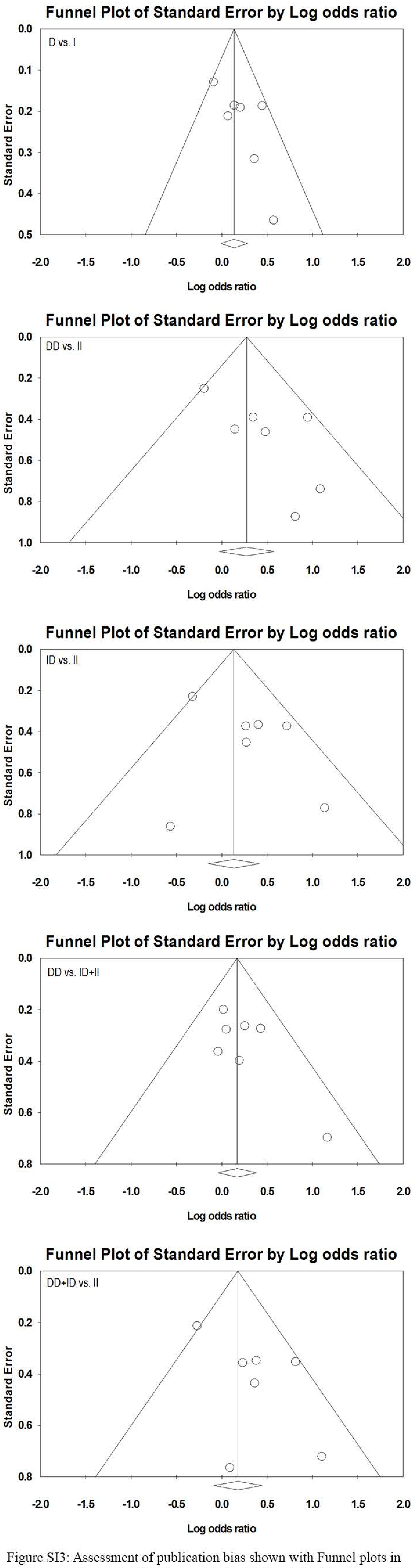

Figure SI3: Assessment of publication bias shown with Funnel plots in studies assaying odds of SLE associated with the ACE I/D polymorphism for sub-group (Caucasian ethnicity population) analysis (Odds ratio against standard error in different genetic models).

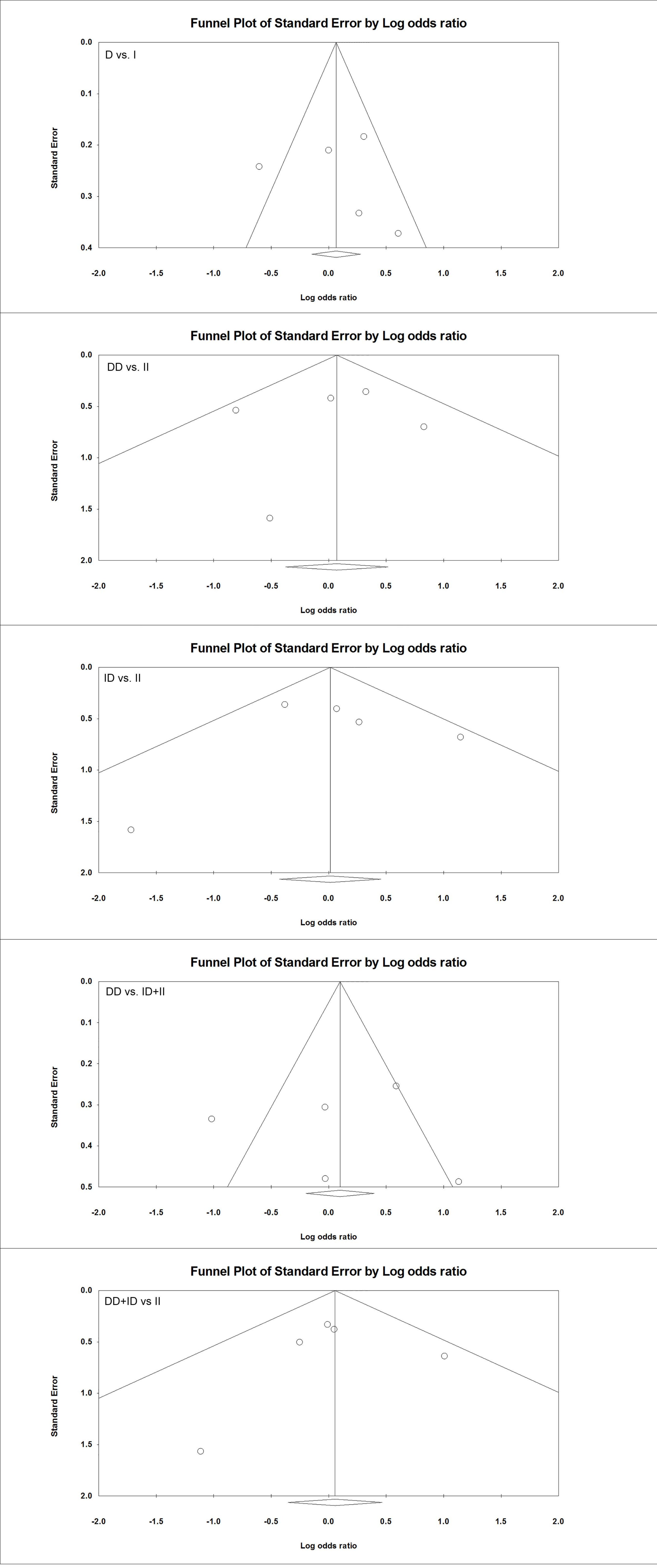

Figure SI4: Assessment of publication bias shown with Funnel plots in studies assaying odds of SLE associated with the ACE I/D polymorphism for sub-group (African ethnicity population) analysis (Odds ratio against standard error in different genetic models).

D vs. I

Study name	Point	Lower limit	Upper limit	Z-Value	p-Value
Pitipakorn et al. 2016	1.228	1.076	1.401	3.049	0.002
Negi et al. 2015	1.208	1.047	1.394	2.596	0.009
Pradhan et al. 2015	1.191	1.038	1.366	2.500	0.012
Topete et al. 2013	1.208	1.052	1.386	2.682	0.007
Salimi et al. 2013	1.191	1.039	1.366	2.502	0.012
Gong et al. 2012	1.180	1.032	1.348	2.430	0.015
Abbas et al. 2012	1.191	1.041	1.364	2.540	0.011
Lian et al. 2012	1.217	1.060	1.396	2.789	0.005
Hussain et al. 2010	1.161	1.028	1.312	2.400	0.016
Rabbani et al. 2008	1.203	1.049	1.379	2.641	0.008
Al-Awadhi et al. 2007	1.217	1.062	1.395	2.827	0.005
El-Shafeey et al. 2005	1.200	1.047	1.375	2.622	0.009
Saeed et al. 2005	1.203	1.049	1.379	2.641	0.008
Sprovieri et al. 2005	1.197	1.043	1.374	2.562	0.010
Douglas et al. 2004	1.211	1.055	1.389	2.723	0.006
Douglas et al. 2004 A	1.205	1.049	1.384	2.636	0.008
Shin et al. 2004	1.212	1.056	1.391	2.737	0.006
Uhm et al. 2002	1.213	1.056	1.393	2.730	0.006
Prkacin et al. 2001	1.195	1.044	1.367	2.584	0.010
Kaufman et al. 2001	1.197	1.042	1.374	2.545	0.011
Kaufman et al. 2001 A	1.218	1.061	1.398	2.803	0.005
Kaufman et al. 2001 B	1.206	1.053	1.383	2.699	0.007
Molad et al. 2000	1.197	1.044	1.372	2.585	0.010
Akai et al. 1999	1.227	1.075	1.401	3.026	0.002
Pullmann et al. 1999	1.190	1.037	1.364	2.485	0.013
Sato et al. 1998	1.197	1.043	1.373	2.557	0.011
Tassiulas et al. 1998	1.231	1.081	1.402	3.139	0.002
Tassiulas et al. 1998 A	1.202	1.046	1.380	2.601	0.009
Guan et al. 1997	1.166	1.027	1.325	2.365	0.018
Combined	1.202	1.052	1.374	2.701	0.007

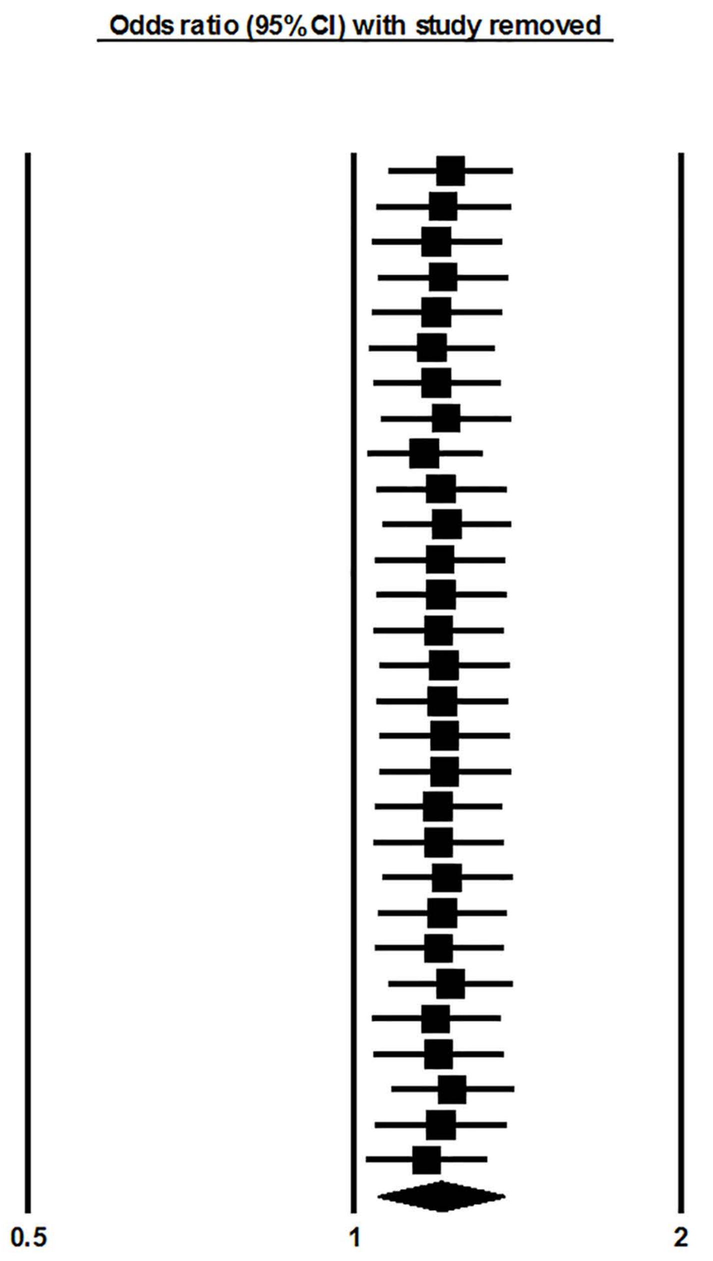

DD vs. II

Study name	Point	Lower limit	Upper limit	Z-Value	p-Value
Pitipakorn et al. 2016	1.424	1.112	1.823	2.802	0.005
Negi et al. 2015	1.356	1.024	1.794	2.128	0.033
Pradhan et al. 2015	1.310	1.005	1.707	1.999	0.046
Topete et al. 2013	1.355	1.035	1.774	2.209	0.027
Salimi et al. 2013	1.316	1.008	1.720	2.018	0.044
Gong et al. 2012	1.280	0.996	1.645	1.932	0.053
Abbas et al. 2012	1.354	1.041	1.761	2.258	0.024
Lian et al. 2012	1.400	1.079	1.817	2.530	0.011
Hussain et al. 2010	1.317	1.012	1.713	2.050	0.040
Rabbani et al. 2008	1.342	1.026	1.755	2.150	0.032
Al-Awadhi et al. 2007	1.384	1.061	1.805	2.400	0.016
El-Shafeey et al. 2005	1.331	1.020	1.735	2.109	0.035
Saeed et al. 2005	1.342	1.026	1.755	2.150	0.032
Sprovieri et al. 2005	1.340	1.024	1.752	2.135	0.033
Douglas et al. 2004	1.362	1.040	1.784	2.246	0.025
Douglas et al. 2004 A	1.345	1.026	1.764	2.143	0.032
Shin et al. 2004	1.370	1.047	1.792	2.298	0.022
Uhm et al. 2002	1.359	1.036	1.784	2.212	0.027
Prkacin et al. 2001	1.335	1.025	1.740	2.141	0.032
Kaufman et al. 2001	1.346	1.026	1.767	2.142	0.032
Kaufman et al. 2001 A	1.382	1.055	1.810	2.346	0.019
Kaufman et al. 2001 B	1.354	1.037	1.768	2.228	0.026
Molad et al. 2000	1.324	1.017	1.725	2.084	0.037
Akai et al. 1999	1.393	1.072	1.810	2.482	0.013
Pullmann et al. 1999	1.311	1.005	1.711	1.997	0.046
Sato et al. 1998	1.340	1.023	1.754	2.128	0.033
Tassiulas et al. 1998	1.393	1.073	1.808	2.487	0.013
Tassiulas et al. 1998 A	1.339	1.023	1.752	2.123	0.034
Guan et al. 1997	1.259	0.989	1.604	1.871	0.061
Combined	1.347	1.038	1.748	2.238	0.025

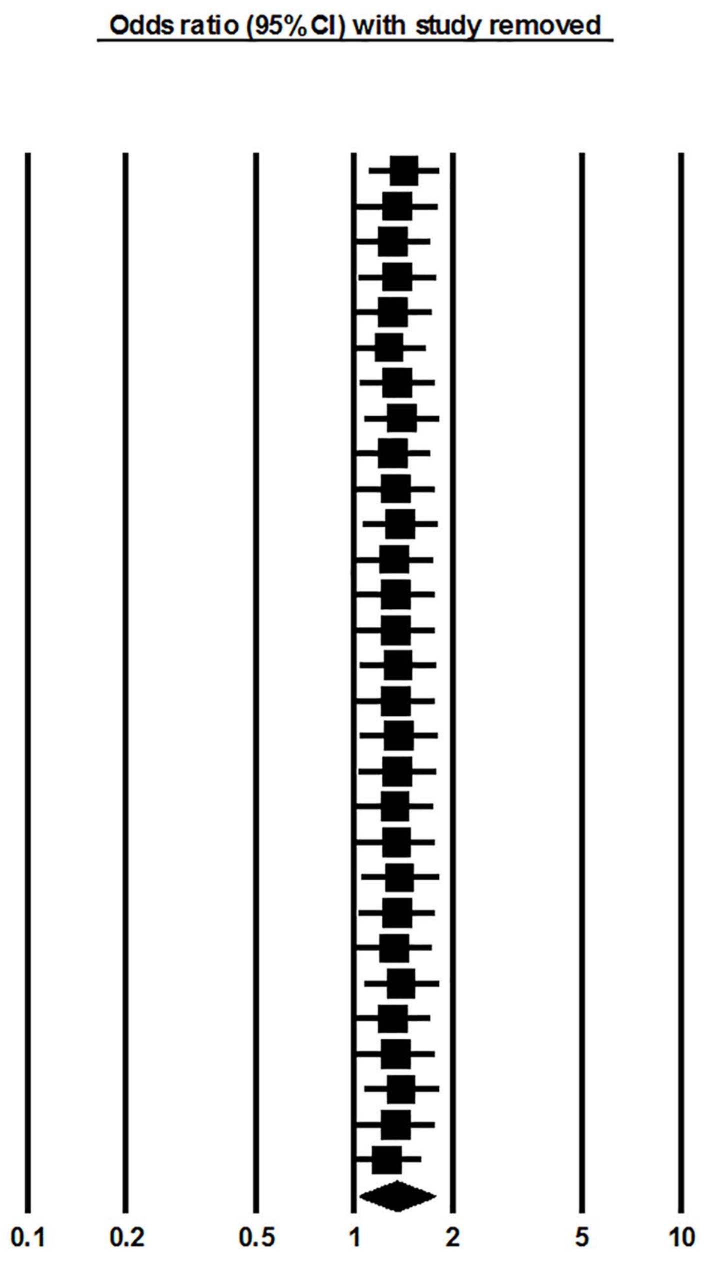

ID vs. II

Study name	Point	Lower limit	Upper limit	Z-Value	p-Value
Pitipakorn et al. 2016	1.072	0.906	1.267	0.809	0.418
Negi et al. 2015	1.095	0.915	1.312	0.993	0.321
Pradhan et al. 2015	1.085	0.916	1.284	0.943	0.346
Topete et al. 2013	1.095	0.922	1.301	1.032	0.302
Salimi et al. 2013	1.098	0.923	1.306	1.051	0.293
Gong et al. 2012	1.087	0.910	1.299	0.923	0.356
Abbas et al. 2012	1.109	0.939	1.309	1.216	0.224
Lian et al. 2012	1.072	0.907	1.267	0.815	0.415
Hussain et al. 2010	1.125	0.960	1.317	1.460	0.144
Rabbani et al. 2008	1.115	0.942	1.321	1.264	0.206
Al-Awadhi et al. 2007	1.124	0.952	1.328	1.378	0.168
El-Shafeey et al. 2005	1.088	0.922	1.284	1.002	0.316
Saeed et al. 2005	1.115	0.942	1.321	1.264	0.206
Sprovieri et al. 2005	1.110	0.936	1.316	1.197	0.231
Douglas et al. 2004	1.102	0.928	1.309	1.111	0.267
Douglas et al. 2004 A	1.090	0.918	1.293	0.982	0.326
Shin et al. 2004	1.099	0.923	1.308	1.061	0.289
Uhm et al. 2002	1.129	0.954	1.337	1.111	0.138
Prkacin et al. 2001	1.109	0.937	1.312	1.209	0.228
Kaufman et al. 2001	1.122	0.948	1.329	1.342	0.179
Kaufman et al. 2001 A	1.133	0.958	1.340	1.461	0.144
Kaufman et al. 2001 B	1.115	0.942	1.319	1.265	0.206
Molad et al. 2000	1.091	0.924	1.288	1.026	0.305
Akai et al. 1999	1.139	0.969	1.340	1.575	0.115
Pullmann et al. 1999	1.080	0.914	1.276	0.901	0.367
Sato et al. 1998	1.082	0.912	1.282	0.904	0.366
Tassiulas et al. 1998	1.098	0.926	1.302	1.072	0.284
Tassiulas et al. 1998 A	1.096	0.924	1.301	1.053	0.292
Guan et al. 1997	1.103	0.926	1.314	1.103	0.270
Combined	1.103	0.934	1.302	1.150	0.250

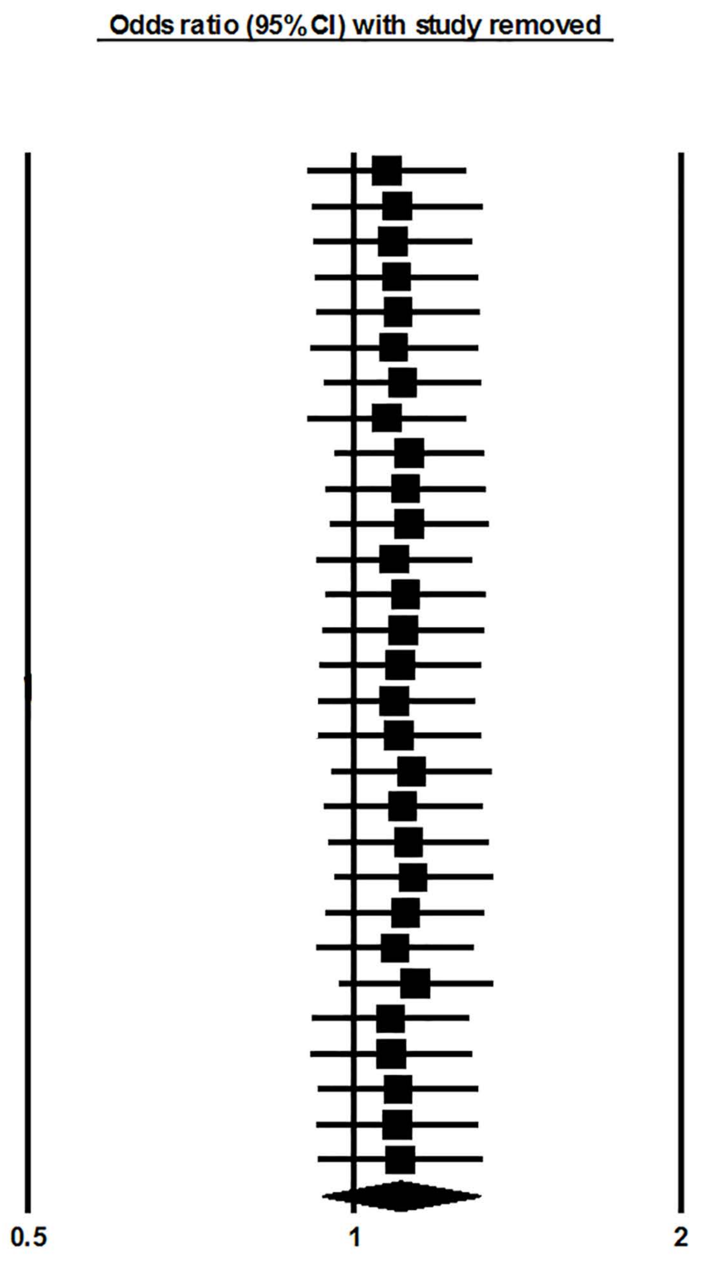

DD vs. ID+II

Study name	Point	Lower limit	Upper limit	Z-Value	p-Value
Pitipakorn et al. 2016	1.412	1.114	1.789	2.857	0.004
Negi et al. 2015	1.351	1.035	1.763	2.216	0.027
Pradhan et al. 2015	1.325	1.023	1.716	2.135	0.033
Topete et al. 2013	1.355	1.047	1.753	2.310	0.021
Salimi et al. 2013	1.315	1.018	1.699	2.098	0.036
Gong et al. 2012	1.294	1.008	1.660	2.023	0.043
Abbas et al. 2012	1.305	1.014	1.681	2.067	0.039
Lian et al. 2012	1.395	1.091	1.784	2.655	0.008
Hussain et al. 2010	1.249	0.991	1.574	1.887	0.059
Rabbani et al. 2008	1.326	1.026	1.713	2.160	0.031
Al-Awadhi et al. 2007	1.356	1.047	1.757	2.304	0.021
El-Shafeey et al. 2005	1.351	1.046	1.745	2.305	0.021
Saeed et al. 2005	1.326	1.026	1.713	2.160	0.031
Sprovieri et al. 2005	1.322	1.022	1.711	2.123	0.034
Douglas et al. 2004	1.356	1.047	1.756	2.305	0.021
Douglas et al. 2004 A	1.352	1.042	1.754	2.273	0.023
Shin et al. 2004	1.362	1.054	1.760	2.364	0.018
Uhm et al. 2002	1.340	1.033	1.737	2.207	0.027
Prkacin et al. 2001	1.315	1.021	1.892	2.123	0.034
Kaufman et al. 2001	1.323	1.020	1.715	2.109	0.035
Kaufman et al. 2001 A	1.356	1.042	1.766	2.263	0.024
Kaufman et al. 2001 B	1.334	1.033	1.723	2.212	0.027
Molad et al. 2000	1.343	1.038	1.738	2.245	0.025
Akai et al. 1999	1.366	1.059	1.762	2.403	0.016
Pullmann et al. 1999	1.332	1.026	1.728	2.156	0.031
Sato et al. 1998	1.344	1.039	1.738	2.253	0.024
Tassiulas et al. 1998	1.401	1.099	1.786	2.722	0.006
Tassiulas et al. 1998 A	1.342	1.033	1.743	2.203	0.028
Guan et al. 1997	1.264	0.995	1.605	1.923	0.054
Combined	1.338	1.042	1.718	2.281	0.023

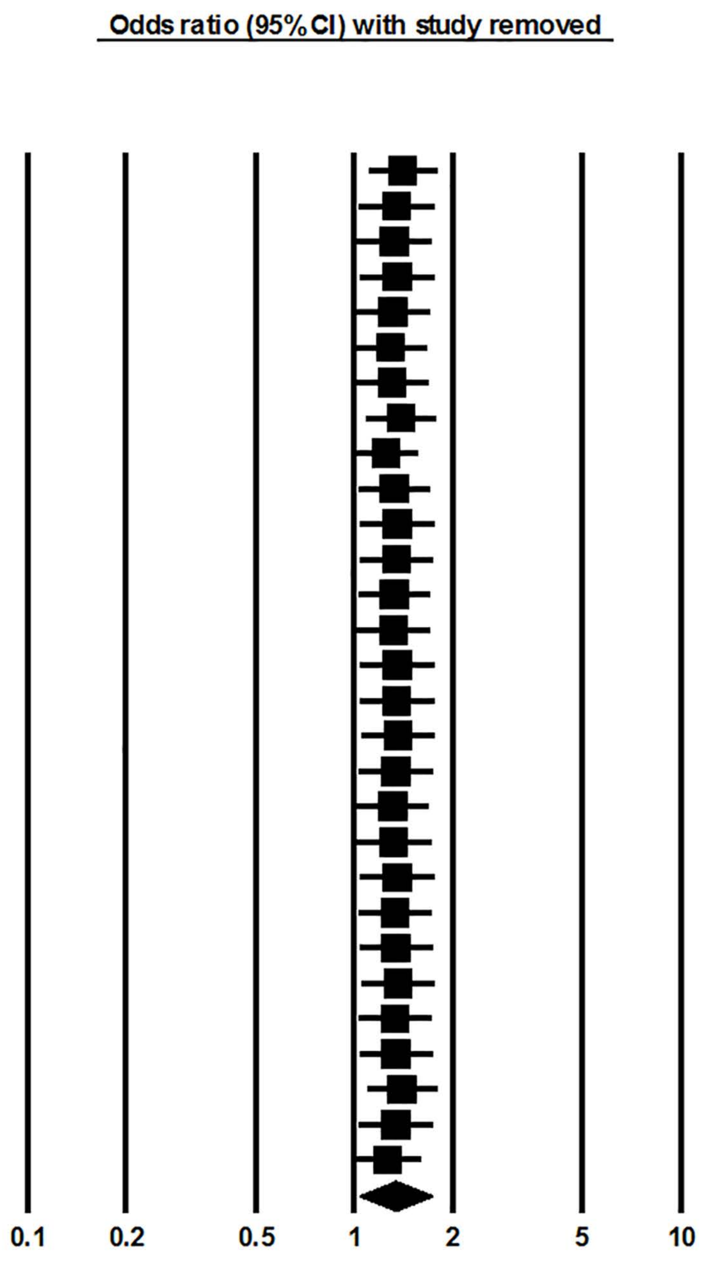

DD+ID vs. II

Study name	Point	Lower limit	Upper limit	Z-Value	p-Value
Pitipakorn et al. 2016	1.203	1.073	1.349	3.171	0.002
Negi et al. 2015	1.198	1.064	1.348	2.995	0.003
Pradhan et al. 2015	1.180	1.056	1.319	2.922	0.003
Topete et al. 2013	1.193	1.067	1.334	3.108	0.002
Salimi et al. 2013	1.186	1.060	1.326	2.974	0.003
Gong et al. 2012	1.147	1.021	1.289	2.308	0.021
Abbas et al. 2012	1.197	1.072	1.336	3.198	0.001
Lian et al. 2012	1.186	1.059	1.330	2.938	0.003
Hussain et al. 2010	1.193	1.068	1.332	3.127	0.002
Rabbani et al. 2008	1.201	1.074	1.342	3.227	0.001
Al-Awadhi et al. 2007	1.212	1.084	1.354	3.383	0.001
El-Shafeey et al. 2005	1.187	1.063	1.326	3.044	0.002
Saeed et al. 2005	1.201	1.074	1.342	3.227	0.001
Sprovieri et al. 2005	1.197	1.071	1.337	3.176	0.001
Douglas et al. 2004	1.198	1.072	1.339	3.187	0.001
Douglas et al. 2004 A	1.188	1.063	1.329	3.034	0.002
Shin et al. 2004	1.200	1.073	1.343	3.181	0.001
Uhm et al. 2002	1.221	1.090	1.367	3.456	0.001
Prkacin et al. 2001	1.195	1.071	1.335	3.171	0.002
Kaufman et al. 2001	1.202	1.075	1.343	3.222	0.001
Kaufman et al. 2001 A	1.235	1.102	1.385	3.633	0.000
Kaufman et al. 2001 B	1.201	1.075	1.342	3.246	0.001
Molad et al. 2000	1.188	1.064	1.327	3.061	0.002
Akai et al. 1999	1.229	1.099	1.375	3.613	0.000
Pullmann et al. 1999	1.175	1.051	1.314	2.840	0.005
Sato et al. 1998	1.180	1.055	1.320	2.890	0.004
Tassiulas et al. 1998	1.201	1.075	1.342	3.247	0.001
Tassiulas et al. 1998 A	1.191	1.066	1.331	3.089	0.002
Guan et al. 1997	1.166	1.041	1.306	2.662	0.008
Combined	1.195	1.070	1.334	3.171	0.002

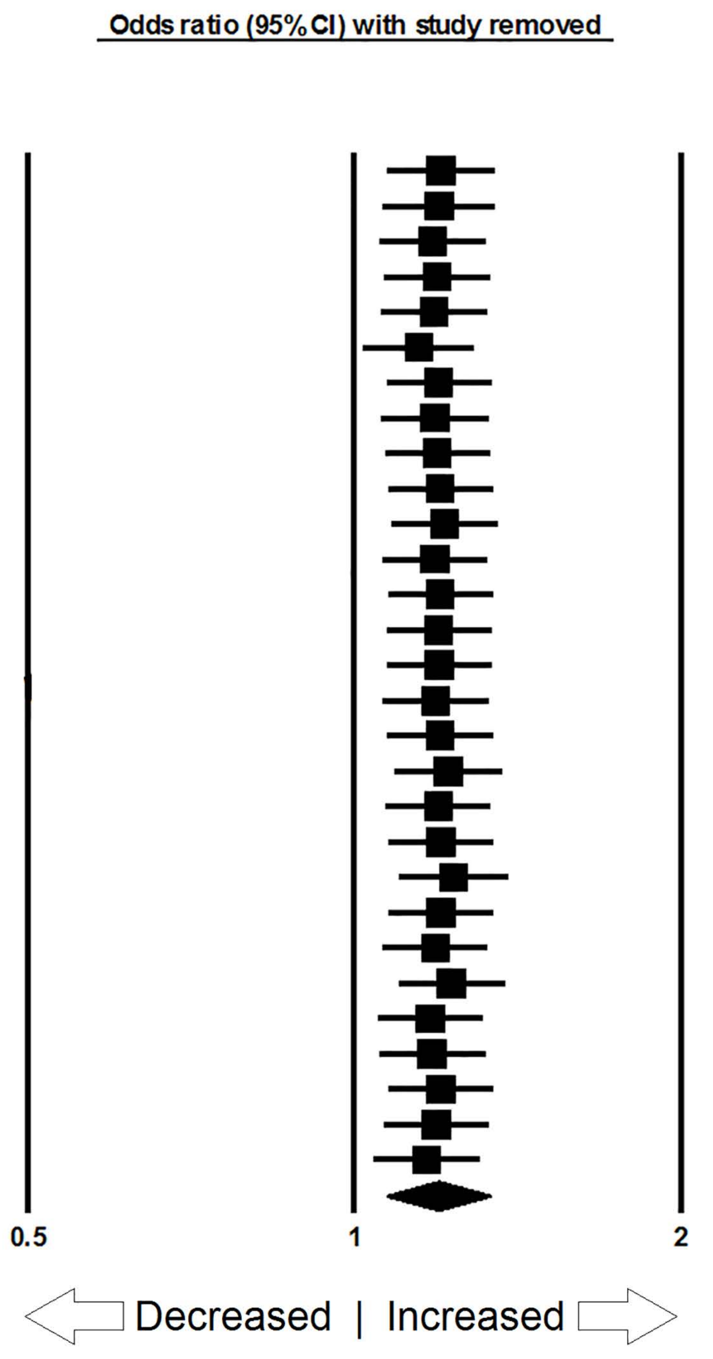

Figure SI5: Sensitivity analysis of ACE I/D polymorphism with overall SLE risk to evaluate the influence of each individual study on the pooled OR by deleting one single study each time for overall analysis (for all the genetic models). Black square represents the value of OR and the size of the square indicates the inverse proportion relative to its variance. Horizontal line is the 95% CI of OR.

D vs. I — Statistics with study removed

Study name	Point	Lower limit	Upper limit	Z-Value	p-Value
Pitipakorn et al. 2016	1.290	1.048	1.587	2.407	0.016
Negi et al. 2015	1.253	0.991	1.585	1.881	0.060
Pradhan et al. 2015	1.220	0.979	1.521	1.771	0.077
Salimi et al. 2013	1.220	0.979	1.521	1.773	0.076
Gong et al. 2012	1.204	0.970	1.494	1.680	0.093
Lian et al. 2012	1.269	1.017	1.583	2.109	0.035
Hussain et al. 2010	1.157	0.960	1.396	1.528	0.126
Rabbani et al. 2008	1.242	0.998	1.545	1.942	0.052
Al-Awadhi et al. 2007	1.271	1.023	1.579	2.163	0.031
Saeed et al. 2005	1.242	0.998	1.545	1.942	0.052
Shin et al. 2004	1.261	1.011	1.572	2.054	0.040
Uhm et al. 2002	1.261	1.010	1.576	2.043	0.041
Akai et al. 1999	1.291	1.048	1.590	2.398	0.017
Sato et al. 1998	1.230	0.987	1.535	1.840	0.066
Guan et al. 1997	1.176	0.962	1.438	1.579	0.114
Combined	1.238	1.005	1.525	2.008	0.045

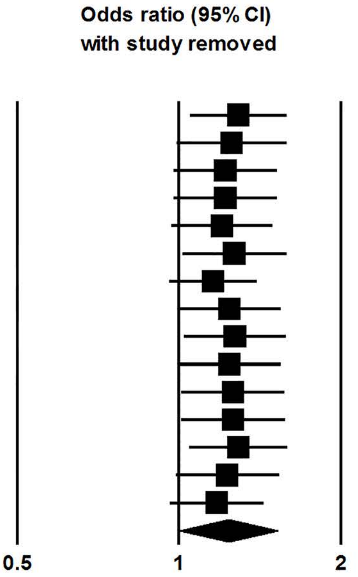

DD vs. II — Statistics with study removed

Study name	Point	Lower limit	Upper limit	Z-Value	p-Value
Pitipakorn et al. 2016	1.514	1.021	2.245	2.065	0.039
Negi et al. 2015	1.382	0.861	2.217	1.340	0.180
Pradhan et al. 2015	1.299	0.843	2.002	1.186	0.236
Salimi et al. 2013	1.312	0.847	2.034	1.216	0.224
Gong et al. 2012	1.263	0.837	1.906	1.111	0.267
Lian et al. 2012	1.465	0.959	2.237	1.767	0.077
Hussain et al. 2010	1.305	0.852	1.999	1.225	0.221
Rabbani et al. 2008	1.358	0.876	2.104	1.369	0.171
Al-Awadhi et al. 2007	1.438	0.933	2.217	1.646	0.100
Saeed et al. 2005	1.358	0.876	2.104	1.369	0.171
Shin et al. 2004	1.411	0.910	2.189	1.537	0.124
Uhm et al. 2002	1.390	0.887	2.176	1.438	0.150
Akai et al. 1999	1.459	0.954	2.229	1.744	0.081
Sato et al. 1998	1.354	0.871	2.106	1.346	0.178
Guan et al. 1997	1.216	0.825	1.792	0.987	0.324
Combined	1.366	0.902	2.070	1.471	0.141

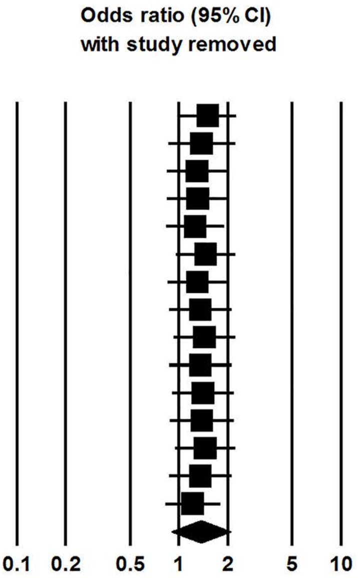

ID vs. II — Statistics with study removed

Study name	Point	Lower limit	Upper limit	Z-Value	p-Value
Pitipakorn et al. 2016	1.047	0.838	1.308	0.402	0.688
Negi et al. 2015	1.076	0.840	1.378	0.581	0.561
Pradhan et al. 2015	1.064	0.851	1.331	0.544	0.586
Salimi et al. 2013	1.083	0.858	1.368	0.670	0.503
Gong et al. 2012	1.065	0.835	1.358	0.506	0.613
Lian et al. 2012	1.047	0.838	1.308	0.407	0.684
Hussain et al. 2010	1.135	0.929	1.388	1.240	0.215
Rabbani et al. 2008	1.114	0.891	1.393	0.950	0.342
Al-Awadhi et al. 2007	1.131	0.909	1.406	1.102	0.270
Saeed et al. 2005	1.114	0.891	1.393	0.950	0.342
Shin et al. 2004	1.085	0.858	1.371	0.681	0.496
Uhm et al. 2002	1.137	0.911	1.420	1.136	0.256
Akai et al. 1999	1.158	0.940	1.426	1.374	0.169
Sato et al. 1998	1.059	0.844	1.329	0.497	0.619
Guan et al. 1997	1.092	0.863	1.382	0.730	0.465
Combined	1.094	0.880	1.360	0.810	0.418

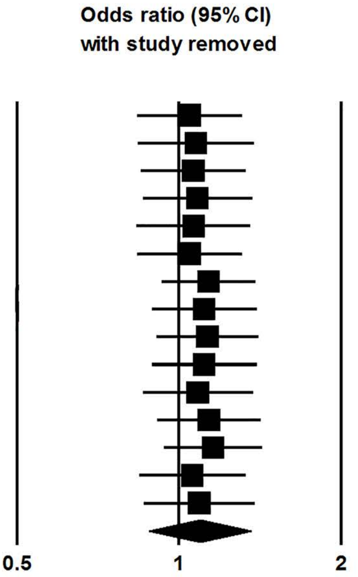

DD vs. ID+II — Statistics with study removed

Study name	Point	Lower limit	Upper limit	Z-Value	p-Value
Pitipakorn et al. 2016	1.615	1.077	2.423	2.319	0.020
Negi et al. 2015	1.470	0.900	2.402	1.538	0.124
Pradhan et al. 2015	1.418	0.890	2.260	1.468	0.142
Salimi et al. 2013	1.396	0.882	2.210	1.425	0.154
Gong et al. 2012	1.362	0.866	2.143	1.336	0.181
Lian et al. 2012	1.571	1.023	2.415	2.062	0.039
Hussain et al. 2010	1.254	0.841	1.869	1.110	0.267
Rabbani et al. 2008	1.416	0.897	2.235	1.495	0.135
Al-Awadhi et al. 2007	1.483	0.930	2.364	1.655	0.098
Saeed et al. 2005	1.416	0.897	2.235	1.495	0.135
Shin et al. 2004	1.497	0.949	2.364	1.734	0.083
Uhm et al. 2002	1.448	0.906	2.313	1.549	0.121
Akai et al. 1999	1.509	0.961	2.370	1.786	0.074
Sato et al. 1998	1.457	0.921	2.306	1.607	0.108
Guan et al. 1997	1.292	0.849	1.964	1.197	0.231
Combined	1.437	0.931	2.217	1.639	0.101

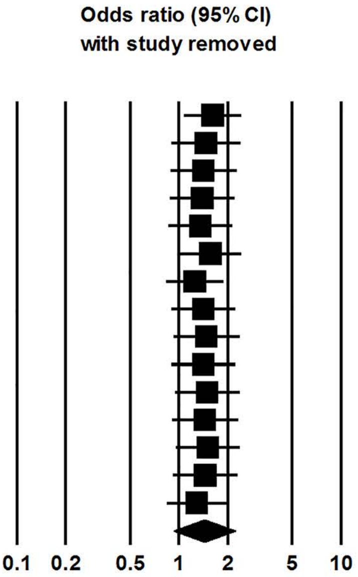

DD+ID vs. II — Statistics with study removed

Study name	Point	Lower limit	Upper limit	Z-Value	p-Value
Pitipakorn et al. 2016	1.199	0.984	1.462	1.803	0.071
Negi et al. 2015	1.190	0.969	1.460	1.661	0.097
Pradhan et al. 2015	1.164	0.971	1.395	1.642	0.101
Salimi et al. 2013	1.174	0.969	1.422	1.637	0.102
Gong et al. 2012	1.146	0.952	1.379	1.441	0.150
Lian et al. 2012	1.178	0.966	1.436	1.622	0.105
Hussain et al. 2010	1.185	0.984	1.428	1.789	0.074
Rabbani et al. 2008	1.204	0.997	1.452	1.933	0.053
Al-Awadhi et al. 2007	1.229	1.029	1.467	2.282	0.023
Saeed et al. 2005	1.204	0.997	1.452	1.933	0.053
Shin et al. 2004	1.199	0.988	1.454	1.838	0.066
Uhm et al. 2002	1.233	1.027	1.481	2.243	0.025
Akai et al. 1999	1.263	1.081	1.476	2.938	0.003
Sato et al. 1998	1.165	0.965	1.406	1.589	0.112
Guan et al. 1997	1.152	0.958	1.385	1.506	0.132
Combined	1.192	0.995	1.428	1.908	0.056

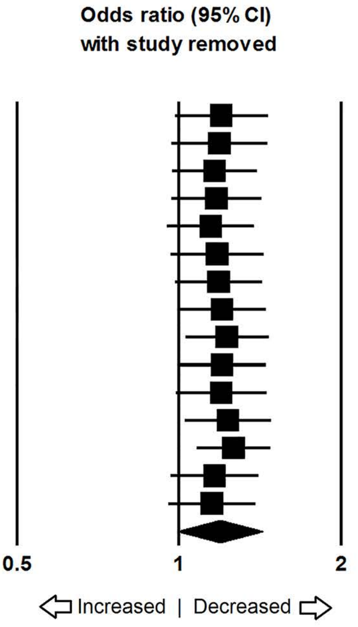

Figure SI6: Sensitivity analysis of ACE I/D polymorphism with TB risk among Asian population to evaluate the influence of each individual study on the pooled OR by deleting one single study each time for subgroup analysis (for all the genetic models). Black square represents the value of OR and the size of the square indicates the inverse proportion relative to its variance. Horizontal line is the 95% CI of OR.

D vs. I

Study name	Point	Lower limit	Upper limit	Z-Value	p-Value
	Statistics with study removed				
Topete et al. 2013	1.156	0.990	1.350	1.838	0.066
Douglas et al. 2004	1.145	0.978	1.342	1.682	0.093
Prkacin et al. 2001	1.133	0.978	1.312	1.662	0.097
Kaufman et al. 2001	1.280	1.072	1.528	2.733	0.006
Molad et al. 2000	1.131	0.974	1.313	1.615	0.106
Pullmann et al. 1999	1.081	0.923	1.266	0.967	0.334
Tassiulas et al. 1998	1.132	0.967	1.325	1.540	0.123
Combined	1.146	0.991	1.324	1.837	0.066

Odds ratio (95% CI) with study removed

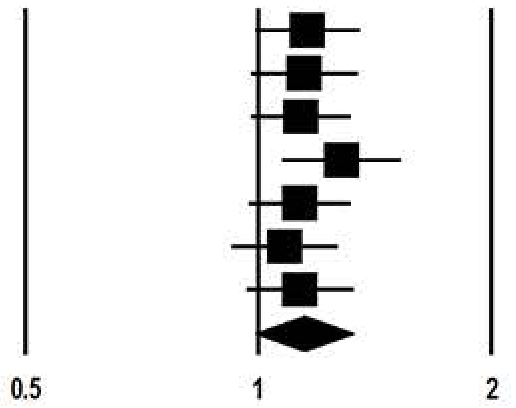

DD vs. II

Study name	Point	Lower limit	Upper limit	Z-Value	p-Value
	Statistics with study removed				
Topete et al. 2013	1.337	0.968	1.846	1.764	0.078
Douglas et al. 2004	1.296	0.932	1.803	1.542	0.123
Prkacin et al. 2001	1.291	0.949	1.757	1.629	0.103
Kaufman et al. 2001	1.750	1.191	2.572	2.851	0.004
Molad et al. 2000	1.266	0.929	1.726	1.493	0.135
Pullmann et al. 1999	1.160	0.834	1.613	0.880	0.379
Tassiulas et al. 1998	1.280	0.928	1.766	1.505	0.132
Combined	1.314	0.971	1.779	1.768	0.077

Odds ratio (95% CI) with study removed

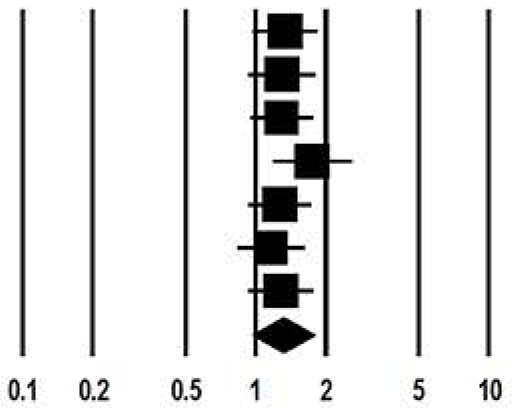

ID vs. II

Study name	Point	Lower limit	Upper limit	Z-Value	p-Value
	Statistics with study removed				
Topete et al. 2013	1.116	0.823	1.512	0.705	0.481
Douglas et al. 2004	1.087	0.801	1.475	0.537	0.591
Prkacin et al. 2001	1.164	0.876	1.548	1.049	0.294
Kaufman et al. 2001	1.525	1.065	2.183	2.302	0.021
Molad et al. 2000	1.101	0.828	1.465	0.662	0.508
Pullmann et al. 1999	1.032	0.761	1.398	0.201	0.841
Tassiulas et al. 1998	1.124	0.836	1.511	0.775	0.438
Combined	1.142	0.862	1.511	0.925	0.355

Odds ratio (95% CI) with study removed

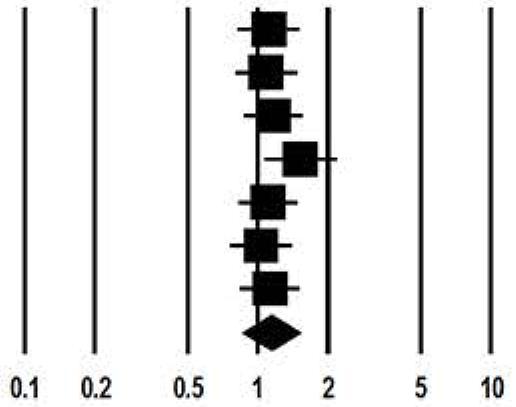

DD vs. ID+II

Study name	Point	Lower limit	Upper limit	Z-Value	p-Value
	Statistics with study removed				
Topete et al. 2013	1.210	0.964	1.519	1.647	0.099
Douglas et al. 2004	1.212	0.957	1.534	1.596	0.111
Prkacin et al. 2001	1.155	0.928	1.437	1.287	0.198
Kaufman et al. 2001	1.265	0.976	1.640	1.778	0.075
Molad et al. 2000	1.182	0.944	1.481	1.457	0.145
Pullmann et al. 1999	1.126	0.889	1.426	0.984	0.325
Tassiulas et al. 1998	1.163	0.917	1.476	1.244	0.214
Combined	1.185	0.954	1.471	1.535	0.125

Odds ratio (95% CI) with study removed

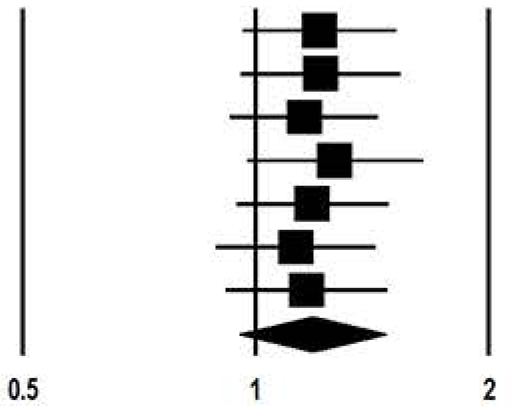

DD+ID vs. II

Study name	Point	Lower limit	Upper limit	Z-Value	p-Value
	Statistics with study removed				
Topete et al. 2013	1.183	0.889	1.574	1.155	0.248
Douglas et al. 2004	1.152	0.865	1.534	0.965	0.335
Prkacin et al. 2001	1.197	0.915	1.566	1.314	0.189
Kaufman et al. 2001	1.608	1.143	2.260	2.730	0.006
Molad et al. 2000	1.154	0.882	1.511	1.045	0.296
Pullmann et al. 1999	1.070	0.804	1.425	0.466	0.641
Tassiulas et al. 1998	1.170	0.886	1.545	1.109	0.267
Combined	1.194	0.917	1.555	1.314	0.189

Odds ratio (95% CI) with study removed

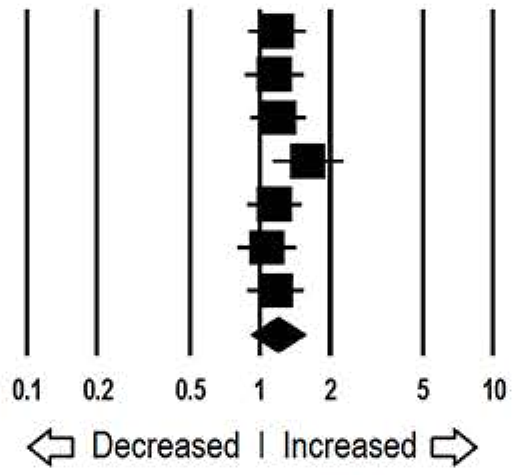

Figure SI7: Sensitivity analysis of ACE I/D polymorphism with TB risk among Caucasian population to evaluate the influence of each individual study on the pooled OR by deleting one single study each time for subgroup analysis (for all the genetic models). Black square represents the value of OR and the size of the square indicates the inverse proportion relative to its variance. Horizontal line is the 95% CI of OR.

D vs. I — Statistics with study removed

Study name	Point	Lower limit	Upper limit	Z-Value	p-Value
Abbas et al. 2012	0.989	0.656	1.492	-0.051	0.959
El-Shafeey et al. 2005	1.048	0.662	1.660	0.201	0.841
Douglas et al. 2004	1.120	0.662	1.895	0.424	0.672
Kaufman et al. 2001	1.015	0.627	1.644	0.060	0.952
Tassiulas et al. 1998	1.260	0.994	1.597	1.914	0.056
Combined	1.083	0.738	1.591	0.408	0.683

Odds ratio (95% CI) with study removed

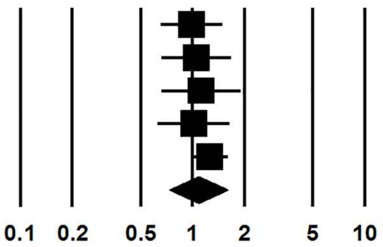

DD vs. II — Statistics with study removed

Study name	Point	Lower limit	Upper limit	Z-Value	p-Value
Abbas et al. 2012	1.086	0.692	1.703	0.359	0.720
El-Shafeey et al. 2005	0.981	0.612	1.571	-0.080	0.936
Douglas et al. 2004	1.096	0.645	1.859	0.338	0.735
Kaufman et al. 2001	0.903	0.507	1.608	-0.348	0.728
Tassiulas et al. 1998	1.297	0.794	2.120	1.038	0.299
Combined	1.073	0.687	1.675	0.309	0.757

Odds ratio (95% CI) with study removed

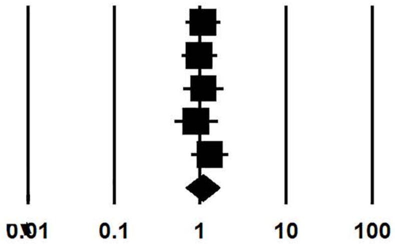

ID vs. II — Statistics with study removed

Study name	Point	Lower limit	Upper limit	Z-Value	p-Value
Abbas et al. 2012	1.050	0.673	1.640	0.216	0.829
El-Shafeey et al. 2005	0.882	0.553	1.407	-0.527	0.598
Douglas et al. 2004	0.988	0.581	1.680	-0.044	0.965
Kaufman et al. 2001	1.295	0.739	2.270	0.903	0.367
Tassiulas et al. 1998	0.960	0.590	1.561	-0.165	0.869
Combined	1.014	0.652	1.575	0.060	0.952

Odds ratio (95% CI) with study removed

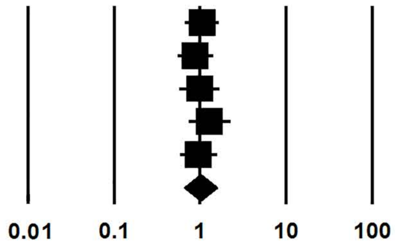

DD vs. ID+II — Statistics with study removed

Study name	Point	Lower limit	Upper limit	Z-Value	p-Value
Abbas et al. 2012	0.898	0.439	1.835	-0.296	0.767
El-Shafeey et al. 2005	1.147	0.501	2.625	0.325	0.745
Douglas et al. 2004	1.160	0.460	2.926	0.315	0.753
Kaufman et al. 2001	0.967	0.425	2.201	-0.080	0.936
Tassiulas et al. 1998	1.474	0.909	2.389	1.574	0.115
Combined	1.110	0.559	2.204	0.298	0.766

Odds ratio (95% CI) with study removed

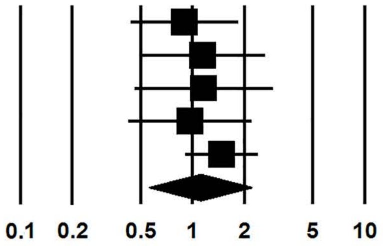

DD+ID vs. II — Statistics with study removed

Study name	Point	Lower limit	Upper limit	Z-Value	p-Value
Abbas et al. 2012	1.080	0.714	1.632	0.363	0.716
El-Shafeey et al. 2005	0.943	0.611	1.455	-0.265	0.791
Douglas et al. 2004	1.061	0.649	1.734	0.234	0.815
Kaufman et al. 2001	1.103	0.651	1.871	0.365	0.715
Tassiulas et al. 1998	1.127	0.718	1.767	0.520	0.603
Combined	1.057	0.702	1.592	0.265	0.791

Odds ratio (95% CI) with study removed

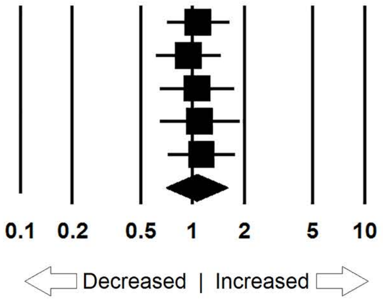

Figure SI8: Sensitivity analysis of ACE I/D polymorphism with TB risk among African population to evaluate the influence of each individual study on the pooled OR by deleting one single study each time for subgroup analysis (for all the genetic models). Black square represents the value of OR and the size of the square indicates the inverse proportion relative to its variance. Horizontal line is the 95% CI of OR.
